# Single-cell profiling reveals context-dependent immune regulation shared by sepsis and systemic lupus erythematosus

**DOI:** 10.3389/fimmu.2026.1892194

**Published:** 2026-09-03

**Authors:** Yan Xu, Hao Wang, Yingchun Hu, Youyu Lan

**Affiliations:** 1 Department of Emergency Medicine, Affiliated Hospital of Southwest Medical University, Luzhou, China; 2 School of Clinical Medicine, Shandong Second Medical University, Weifang, Shandong, China; 3 Department of Rheumatology and Immunology, Affiliated Hospital of Southwest Medical University, Luzhou, China

**Keywords:** sepsis, SIGLEC 1, single-cell sequencing, systemic lupus erythematosus, TRIM25

## Abstract

**Objective:**

To characterize shared immune-associated programs between sepsis and systemic lupus erythematosus (SLE), define the cellular context of prioritized candidate genes, and functionally evaluate SIGLEC1 in macrophage inflammatory responses.

**Methods:**

Public transcriptomic datasets were analyzed to identify common differentially expressed genes using the limma package. Shared candidate genes were characterized using protein-protein interaction and enrichment analyses, and prioritized candidates were subsequently selected using an integrated multi-dimensional evidence framework combining discovery-stage differential expression and orthogonal supporting evidence.Single-cell RNA sequencing (scRNA-seq) analysis was performed on five in-house samples (normal, SIRS, and sepsis) and integrated with external datasets (GSE167363 for sepsis; GSE162577 and GSE136035 for SLE) using Harmony. Cell-type distribution, gene expression patterns, and pathway activity were systematically evaluated. Immune infiltration, protein-level association, and discriminatory performance between disease cohorts and healthy controls was further assessed using external cohorts. Finally, qPCR validation and SIGLEC1 loss-of-function experiments were performed in THP-1-derived macrophages, followed by assessment of inflammatory cytokine production by ELISA and macrophage activation phenotype by flow cytometry.

**Results:**

A total of 49 shared candidate genes were identified in the initial discovery analysis. Using an integrated prioritization framework, TRIM25 and SIGLEC1 ranked third and fourth, respectively, among the 49 candidates and were selected for further investigation based on their high overall prioritization and complementary evidence profiles.Subsequent analyses revealed disease- and cohort-dependent expression patterns of these genes. Single-cell analysis indicated that SIGLEC1 expression was primarily confined to monocyte/macrophage populations, while TRIM25 was more widely expressed among various immune cells. Sepsis samples were characterized by innate immune cell enrichment, while SLE samples showed increased adaptive immune cell proportions. Pathway analysis demonstrated shared activation of inflammatory signaling but distinct immune programs between diseases. Both genes exhibited potential discriminatory capacity between disease cohorts and healthy controls, while experimental validation supported the inducible expression patterns of both genes under disease-associated stimulation. Furthermore, SIGLEC1 knockdown attenuated LPS-induced IL-6 and TNF-α production and reduced the CD86-positive macrophage phenotype, providing functional evidence for its involvement in macrophage inflammatory responses.

**Conclusion:**

SIGLEC1 and TRIM25 are context-dependent immune signatures in sepsis and SLE, with loss-of-function evidence supporting SIGLEC1 in macrophage inflammatory responses.

## Introduction

Although sepsis and systemic lupus erythematosus (SLE) have distinct etiological backgrounds and clinical trajectories, increasing evidence suggests that both diseases involve partially overlapping innate immune regulatory programs, particularly interferon-associated responses, myeloid-cell activation, and dysregulated inflammatory signaling. Sepsis is a systemic inflammatory response syndrome triggered by infection, marked by an excessive inflammatory reaction and immune system dysregulation. This dysregulation can result in multi-organ failure, the primary cause of mortality in sepsis patients (1). In sepsis, overactivation of the immune system leads to a cytokine storm that further exacerbates the inflammatory response (2).

Conversely, SLE is a chronic autoimmune disorder characterized by abnormalities in both the innate and adaptive immune systems. Patients with SLE often exhibit a loss of immune tolerance to their own antigens, leading to the production of autoantibodies and multiorgan damage (3). In the pathophysiology of SLE, overproduction of inflammatory factors and abnormal activation of the immune system are key factors (4). Furthermore, SLE and sepsis both exhibit immune system abnormalities. Studies have shown that metabolic reprogramming of immune cells in SLE patients may lead to immune imbalance and triggering of autoimmune inflammation (4). Similarly, immune dysregulation in sepsis is associated with altered immune metabolism, which may affect disease progression and prognosis (5).

Commonalities between the two diseases also include their complex impact on the immune system. Both SLE and sepsis involve multifaceted dysregulation of the immune system, including cytokine imbalances, abnormal immune cell function, and loss of immune tolerance (6). These common pathologic mechanisms provide potential research directions and therapeutic targets for understanding and treating these two complex diseases (7). Although sepsis and systemic lupus erythematosus (SLE) arise from distinct pathological triggers, accumulating evidence suggests that these disorders share partially overlapping immune regulatory mechanisms. Dysregulated innate immune activation, abnormal cytokine networks, interferon-associated responses, and alterations in immune-cell homeostasis represent common features involved in the progression of various inflammatory diseases. Recent studies have highlighted the importance of conserved inflammatory pathways and immune regulatory networks across distinct disease contexts, suggesting that comparative analyses of different inflammatory disorders may provide insights into shared molecular mechanisms and potential biomarkers (8–10).

Advancements in high-throughput genomics technologies have significantly advanced the study of molecular mechanisms in sepsis and SLE. Gene expression profiling is extensively utilized to identify crucial molecules and pathways in various diseases. Recent studies indicate that convergent immune programs, including type I interferon signaling, pattern-recognition receptor activation, and monocyte/macrophage-mediated immune regulation, may contribute to inflammatory disorders with distinct etiologies. However, whether specific immune regulatory components are shared between sepsis and SLE remains incompletely understood.

**Figure 1:**
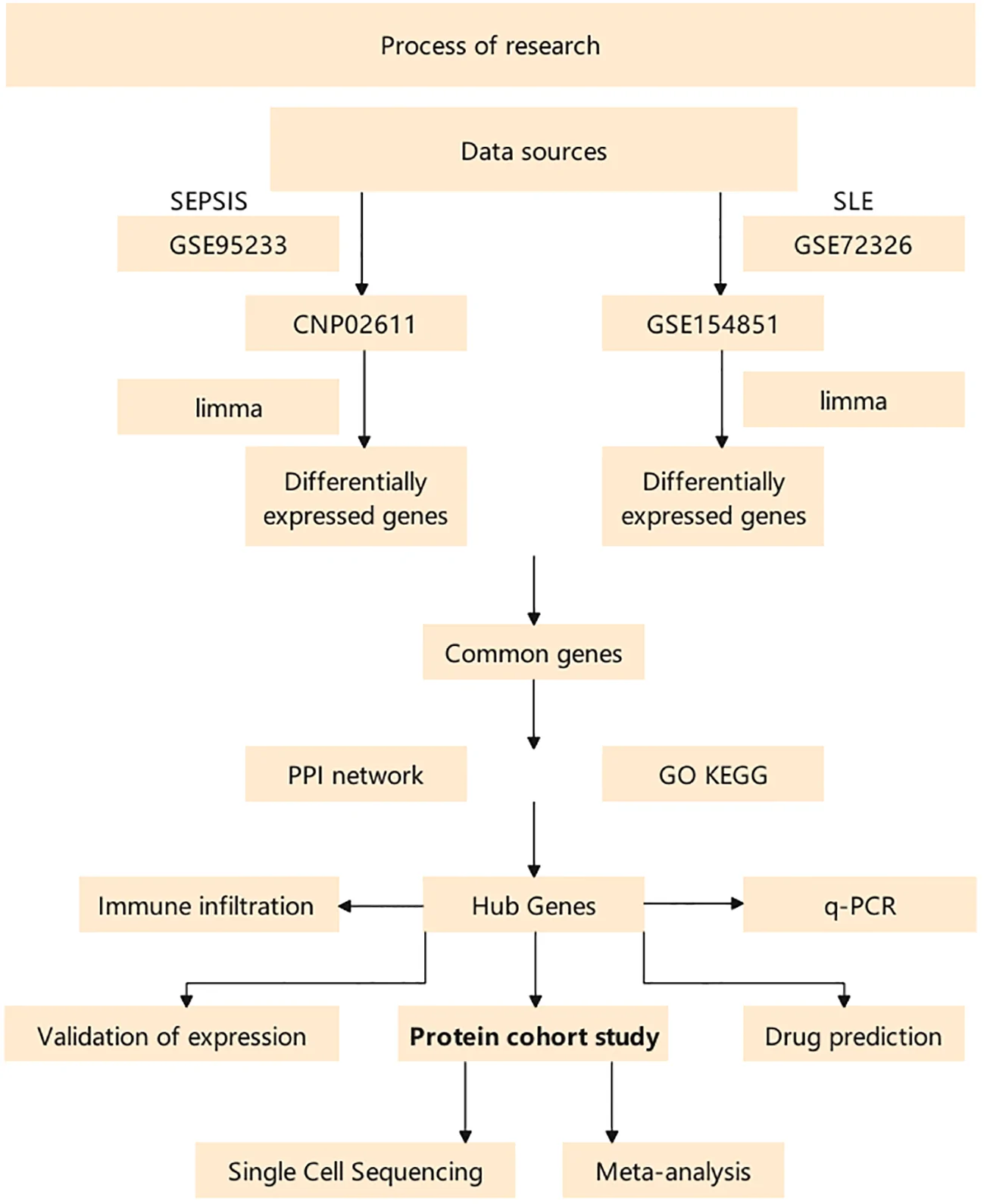
Flow chart of this study. To identify potential targets of sepsis and SLE.

This research investigates shared molecular mechanisms between sepsis and SLE using various bioinformatics tools and high-throughput data integration. We identified shared differentially expressed genes (DEGs) in both diseases through differential expression analysis and characterized their interaction network using protein-protein interaction (PPI) analysis. Candidate genes were subsequently prioritized using an integrated framework combining discovery-stage quantitative ranking and orthogonal supporting evidence. We further investigated the cellular localization and disease-associated expression patterns of prioritized candidates SIGLEC1 and TRIM25 using estimated immune-cell abundance analysis and single-cell transcriptomic sequencing. In addition, we integrated single-cell transcriptomic analysis with external validation cohorts to investigate the cellular localization and disease-associated characteristics of key genes (SIGLEC1 and TRIM25). These studies aim to uncover the shared pathogenesis of sepsis and SLE, offering scientific evidence for the identification of candidate biomarkers and future mechanistic investigations. **Figure 1** illustrates the study’s progression.

## Methodology

### Data sources

This study utilized publicly accessible gene expression datasets: GSE72326 (11), GSE154851 (12), CNP02611, and GSE95233 (13). The CNP02611 dataset includes samples from the Affiliated Hospital of Southwest Medical University, comprising 23 sepsis patients and 10 healthy volunteers admitted between February 2019 and December 2020. Their samples were collected and analyzed within 24 hours of admission. Inclusion criteria were: (1) sepsis patients in the acute ICU, (2) sepsis diagnosed per Sepsis 3.0 criteria (infection + ΔSOFA score ≥2) by the Society of Intensive Care Medicine and the European Society of Intensive Care Medicine, (3) age 16-65, and (4) consented participants or their legal representatives. Exclusion criteria included patients with a history of organ failure, immune system disease, hematologic disease, or those unwilling to participate in the study. The hospital’s Ethics Committee approved the study plan and informed consent. All samples were obtained with informed consent from the patients and their families (ky2018029). The clinical trial is registered under ChiCTR1900021261. The gene expression profiling datasets GSE154851 and GSE72326, available in the Gene Expression Omnibus (GEO), are primarily utilized for systemic lupus erythematosus (SLE) research. GSE154851 includes samples from 38 SLE patients and 32 healthy controls, and it is frequently employed for differential gene expression analysis, gene enrichment analysis, and developing disease risk prediction models.GSE72326 is a longitudinal dataset featuring samples collected at various time points, identifying crucial genes and immune signatures linked to SLE via differential gene expression analysis, weighted gene co-expression network analysis (WGCNA), and immune infiltration analysis. These two datasets provide important support for studying the molecular mechanisms of SLE and identifying potential biomarkers. In addition, GSE95233 investigated gene expression and its prognosis-related molecules in patients with septic shock. These datasets provide reliable basic data support for this study. All experimental methods adhered to relevant guidelines and regulations.

### Differential screening

This study conducts differential gene analysis to identify shared differentially expressed genes (DEGs) in SLE and sepsis, aiming to uncover potential molecular mechanisms underlying both diseases. This study analyzed two gene expression datasets, CNP02611 and GSE154851, using R version 4.4.3. The normalized gene expression matrix was downloaded and preprocessed by the GEOquery package, and the limma package was used to perform the differential gene screening, which was performed using the screening criteria of |log2FoldChange| ≥ 1 and the corrected P-value (adj.P. Val) < 0.05. The ggplot2 package was utilized to create volcano plots, illustrating the distribution of significantly up-regulated, down-regulated, and non-significantly differentiated genes. Subsequently, the significantly up-regulated genes in the two datasets were extracted, and the intersection was taken to obtain the common differential genes of the two diseases (14). Heatmaps illustrating the expression patterns of common genes in the CNP02611 and GSE154851 datasets were generated using the pheatmap package.

Construction of the PPI network and subsequent enrichment analysis.

To investigate the functions and interactions of common differential genes, we constructed a protein-protein interaction (PPI) network using the STRING database (https://string-db.org) (15). The 49 identified differential genes were analyzed using the STRING database with ‘Homo sapiens’ as the species, a minimum interaction confidence of 0.7, and unconnected nodes excluded to derive the interaction network data. Subsequently, the network was exported for visualization and descriptive assessment of interaction patterns among the shared candidate genes. The Metascape database (https://metascape.org) facilitated Gene Ontology (GO) functional annotation and Kyoto Encyclopedia of Genes and Genomes (KEGG) pathway analysis for common differential genes in this study (16, 17). The common gene list was uploaded to the Metascape platform for GO and KEGG enrichment analysis using Homo sapiens as the species background. A P-value threshold of < 0.05 was applied, and the most representative gene set entries were visualized. GO annotations included biological process (BP), cellular component (CC), and molecular function (MF), while KEGG annotations encompassed gene ontology and pathways. KEGG analysis was used to reveal the functional enrichment of common genes in signaling pathways (18, 19). By visualizing the enrichment results with bar graphs and pathway annotations, the possible biological roles of common differential genes in disease development were further clarified. The above analytical methods provide important reference data for exploring the common molecular mechanisms of SLE and Sepsis.

### Candidate gene prioritization strategy

To minimize potential selection bias, all 49 shared candidate genes were evaluated using a transparent integrated prioritization framework. Discovery-stage evidence was quantified using differential-expression magnitude and statistical significance across the SLE and sepsis discovery datasets. Orthogonal supporting evidence incorporated cross-cohort reproducibility, single-cell expression characteristics, discriminatory performance, and available protein-level evidence. The orthogonal evidence score was calculated as the mean of the cross-cohort, single-cell, ROC, and protein-support scores, and the overall prioritization score combined discovery-stage and orthogonal evidence with equal weighting. PPI metrics were retained as supplementary network information and were not incorporated into the overall prioritization score.

Based on this predefined framework, TRIM25 and SIGLEC1 ranked third and fourth, respectively, among the 49 candidates (**Supplementary Table 1**). Importantly, these genes were not selected because of PPI connectivity or because they represented the top two candidates according to a single metric. Rather, they were retained as high-priority representative candidates with complementary evidence profiles. TRIM25 showed concordant positive differential expression in both discovery datasets together with additional cross-cohort and single-cell support, whereas SIGLEC1 showed strong orthogonal support, particularly from single-cell localization, discriminatory performance, and protein-level evidence.

### Estimated immune-cell abundance analysis

We estimated immune-cell abundance and examined the correlation between key genes and immune-cell enrichment scores using the CNP02611 and GSE154851 datasets to investigate the roles of key genes in the immune microenvironment. Immune-cell abundance was estimated using single-sample gene set enrichment analysis (ssGSEA), which quantitatively evaluates immune cell abundance by calculating enrichment scores for specific gene sets in each sample (20). The LM22 immune signature was selected because it provides predefined immune-cell-specific gene sets covering multiple immune populations, including myeloid and lymphoid subsets, and has been widely used for estimating immune-cell-associated transcriptional patterns in human peripheral blood transcriptomic datasets. Given the nature of ssGSEA analysis, the resulting scores represent relative enrichment of immune-cell-related signatures rather than absolute cellular proportions. First, specific gene sets for 28 immune cells were obtained from immune-related gene sets, such as LM22, and the normalized gene expression matrices were enriched using the gsva function of the GSVA package to generate an enrichment score matrix for immune cells. Subsequently, heatmaps were generated using the pheatmap package to show the infiltration patterns of immune cells in different samples and the differences between SLE and Sepsis states. Next, key genes (SIGLEC1 and TRIM25) were analyzed for correlation with immune cells. The Spearman correlation coefficients and p-values between key gene expression levels and immune cell enrichment scores were determined using the cor. test function to evaluate correlation strength and significance. The ggplot2 package was utilized to create correlation lollipop charts for visualizing the positive or negative relationships between key genes and immune cells, histograms to depict correlation coefficient strengths, and scatter plots to illustrate the distribution trends of gene expression alongside immune cell enrichment scores. With this analysis method, we systematically characterized the associations between key gene expression patterns and immune-cell enrichment profiles in SLE and sepsis, which provided important data to support the understanding of their biological functions in the disease microenvironment. Considering that peripheral blood transcriptomic profiles may be influenced by variations in leukocyte composition, we further performed cell-composition-adjusted analyses. Immune-cell infiltration scores estimated by ssGSEA were incorporated into multivariable linear regression models to evaluate whether the associations between candidate gene expression and disease status were independent of immune-cell composition changes. SIGLEC1 and TRIM25 expression levels were used as dependent variables, while disease status and major immune-cell infiltration scores were included as explanatory variables.

### Protein cohort study

To explore the association between plasma protein levels of key genes and health-related phenotypes, this study utilized the Proteome-Phenome Atlas database (https://proteome-phenome-atlas.com/) (21).The database, derived from UK Biobank data, includes proteomic and phenotypic information from around 50,000 participants, detailing the associations between approximately 3,000 plasma proteins and over 1,000 health-related phenotypes.The Proteome-Phenome Atlas database, with its large data coverage, multiple phenotypic associations, and open access, is an important resource for studying protein-disease associations.In the Proteome-Phenome Atlas database, key gene names (SIGLEC1 and TRIM25) were entered to retrieve plasma protein association data corresponding to the genes.Statistical data, including hazard ratios (HR), 95% confidence intervals (CI), and significant P-values, were collected for protein associations and analyzed for links to target diseases (SLE and Sepsis) and immune-related phenotypes.The extracted association results were organized into a structured data table, including information such as protein name, phenotype, HR, 95% CI, P value and sample size.The data were organized in R (version 4.4.3), and the tidyverse package was used for data cleaning and structure optimization.Based on the extracted association information and the phenotypic effects of the key genes, the significantly associated phenotypes were screened out (P value < 0.05), and the association strength and significance of the key genes were calculated.The analysis was focused on the results related to systemic lupus erythematosus (SLE) and sepsis, while focusing on other phenotypes related to immune function.Forest plots were created with the ggplot2 package in R to illustrate the association strength (HR) and significance between health phenotypes and protein levels.The forest plots visualize the interval estimates of HR and their directionality, further highlighting the results of significant associations.Through the above methods, this study comprehensively integrated the information from the Proteome-Phenome Atlas database to provide an in-depth analysis of the associations between key genes and multiple health-related phenotypes, and provided strong basic data to support the exploration of the roles of SIGLEC1 and TRIM25 in immune-related diseases.

### Expression validation of key genes

To explore the expression differences of key genes SIGLEC1 and TRIM25 in systemic lupus erythematosus (SLE) and sepsis and their diagnostic efficacy, this study was analyzed using external datasets GSE95233 and GSE72326. Normalized expression data were obtained from the Gene Expression Omnibus (GEO) database and analyzed using R (version 4.4.3). The expression levels of SIGLEC1 and TRIM25 were analyzed across disease groups (SLE and Sepsis) and a normal control group (NC). Differentially expressed genes were identified using the linear model from the limma package, with significance determined by adjusted P-values (P.Val < 0.05). Histograms were used to visualize the results, displaying the mean and standard error of gene expression between the two groups, along with significance annotations indicating the level of difference. The discriminatory performance of SIGLEC1 and TRIM25 between disease cohorts and healthy controls was assessed by generating ROC curves and calculating the AUC with the pROC package (22). The `roc` function performed ROC curve analysis, utilizing AUC values and their 95% confidence intervals (CIs) to evaluate their ability to distinguish disease cohorts from healthy controls. The ROC curves of the GSE95233 and GSE72326 datasets were plotted separately, and the AUC values and CIs were labeled in the plots to visualize the diagnostic efficacy. By the above methods, the expression characteristics of key genes in diseases and their diagnostic efficacy were systematically evaluated in this study, which provides an important basis for their research as potential biomarkers.

### Single-cell sequencing

Single-cell RNA sequencing was performed using the 10x Genomics platform, which is based on microfluidic technology that encapsulates individual cells into droplets and labels each cell and transcript with a unique Cell Barcode and Unique Molecular Identifier (UMI). After cell lysis and reverse transcription in droplets, cDNA libraries were created and sequenced using the Illumina platform. Raw sequencing data were processed with Cell Ranger (10x Genomics) for genome alignment, UMI de-duplication, and gene expression quantification. Quality control measures were implemented to eliminate low-quality, doublet, and apoptotic cells. Cells were selected for downstream analysis if their gene and UMI counts fell within the mean ± 2 standard deviations and their mitochondrial gene proportions were below 20%. After dimensionality reduction, cell clustering was conducted using the shared nearest neighbor (SNN) algorithm, and cell types were annotated with the SingleR package utilizing the Human Primary Cell Atlas (HPCA) reference dataset (23).

In this study, five in-house samples (including normal controls, SIRS, and sepsis patients) were generated and analyzed. To improve robustness and generalizability, these self-generated data were further integrated with a publicly available sepsis scRNA-seq dataset (GSE167363). In addition, two independent SLE datasets (GSE162577 and GSE136035) were incorporated for cross-disease validation. The Harmony algorithm was applied for data integration and batch effect correction after principal component analysis (PCA), and visualization was achieved using uniform manifold approximation and projection (UMAP). The expression patterns of key genes, such as SIGLEC1 and TRIM25, were analyzed across various cell populations, with differential expression evaluated using the MAST method. Pathway activity at the single-cell level was assessed using gene set variation analysis (GSVA) to identify functional differences between sepsis and SLE. These analyses enabled precise delineation of the cellular localization and expression patterns of key genes. To further avoid potential bias caused by treating individual cells as independent biological replicates, patient-level pseudobulk analysis was additionally performed. Raw counts were aggregated according to individual sample identity and cell type, and differential expression analysis was conducted using edgeR quasi-likelihood generalized linear models with each patient/sample considered as the biological replicate.

### Meta-analysis

To systematically evaluate the expression differences of candidate genes such as TRIM25 and SIGLEC1 across various disease states, a comprehensive meta-analysis was performed based on publicly available datasets. Gene expression profiles were sourced from the GEO database, selecting datasets based on these criteria: (1) human peripheral blood samples; (2) inclusion of both disease and control groups with standardized expression values, sample sizes, and standard deviations; and (3) datasets related to sepsis, systemic inflammatory response syndrome (SIRS), or COVID-19.

Specifically, studies comparing sepsis survivors and non-survivors (GSE33118, GSE95233, GSE185263), normal controls and sepsis patients (including GSE154851, GSE28735, GSE95233, GSE9963, GSE26423, GSE132143, GSE17755, GSE185263, GSE149614, GSE134347, GSE101159, GSE232753, GSE6603, GSE243217, GSE154927), SIRS versus sepsis (GSE134347, GSE9963, GSE236713, GSE243217, GSE154916, GSE134342, GSE212379, GSE6603), pediatric sepsis (GSE4607, GSE8121, GSE26440, GSE72812, GSE154227, GSE11921, GSE13904), and COVID-19 versus healthy individuals (GSE243217, GSE189990, GSE161351) were included.

The effect size for all comparisons was represented by the standardized mean difference (SMD). Pooled SMDs were calculated using both fixed-effect and random-effects models, with study heterogeneity evaluated through Cochran’s Q test and the I² statistic. In cases of significant heterogeneity (I² > 50% or p < 0.10), the random-effects model was prioritized. Forest plots were created to display individual and combined effect sizes, including 95% confidence intervals.To minimize potential bias caused by repeated sampling, longitudinal samples, and inconsistent comparison definitions, we performed an additional harmonized reanalysis using independent baseline cohorts where available. Effect directions were uniformly defined as disease group minus reference group, and detailed cohort-level information, including sample type, comparison design, effect size, and statistical significance, was summarized in **Supplementary Table 2**.

The analyses utilized the R packages meta and metafor. Subgroup analyses were performed for each candidate gene across five comparison groups (1): sepsis survivors vs non-survivors (2), normal vs sepsis (3), SIRS vs sepsis (4), pediatric normal vs pediatric sepsis, and (5) normal vs COVID-19. Results were reported with confidence intervals and study-specific weights to ensure transparency and comparability across disease conditions. These subgroup analyses were designed to evaluate whether gene expression patterns were conserved or context-dependent across different inflammatory conditions. To reduce potential clinical confounding, additional screening criteria were applied during dataset selection. GEO datasets were carefully reviewed for available clinical information, including concurrent infection, corticosteroid exposure, immunosuppressive treatment, and sampling time. Datasets were excluded from pooled analysis when these factors could not be separated from the disease condition or when they were likely to introduce substantial bias into the comparison. When available, baseline or pretreatment samples were preferentially selected. These criteria were applied to minimize the influence of treatment-related and infection-related alterations in immune-cell composition and inflammatory transcriptional signatures.

### Key gene expression validation

#### 1 THP-1 cell culture and macrophage differentiation

THP-1 cells (ATCC^®^ TIB-202™) were cultured in RPMI-1640 medium with 10% FBS, 1% penicillin/streptomycin, and 2 mM L-glutamine at 37 °C and 5% CO_2_. Cell density was maintained at 0.5–1 × 10^6^ cells/mL, with passages performed every 48–72 hours. Monocyte differentiation into macrophages was achieved by seeding cells at 5 × 10^5^ cells per well in 6-well plates and treating them with 160 nM phorbol esters (PMA) for 24 hours. After differentiation, cells were gently washed twice with PBS, and complete medium without PMA was added for 24 hours to eliminate residual effects.

#### 2 Disease model construction

The differentiated THP-1 macrophages were divided into three groups for treatment:

Control group: treated with an equal volume of PBS for 24 hours;

The sepsis group was exposed to 1 μg/mL ultra-pure lipopolysaccharide (LPS, E. coli O111 B4) for 24 hours to mimic sepsis-induced inflammation.

SLE-associated immune-metabolic regulation group: treated with 10 μM 4-octyl itaconate (4-OI) for 24 hours to investigate itaconate-associated immunometabolic responses relevant to SLE-related immune dysregulation (24). 3. RNA extraction followed by quantitative real-time PCR analysis. The selected LPS concentration and incubation duration were based on previous THP-1 macrophage activation studies demonstrating that 1 μg/mL LPS stimulation for 24 hours can induce robust inflammatory transcriptional responses while maintaining cellular viability. Similarly, the 4-OI treatment condition was selected based on previous studies investigating itaconate-mediated immunometabolic regulation in macrophage models. These conditions were designed to induce disease-associated immune responses and metabolic regulation rather than to fully reproduce the complex pathological environments of sepsis or systemic lupus erythematosus (25–27).

Total RNA was extracted using TRIzol reagent, and purity was assessed by spectrophotometry (A260/A280 ratio 1.8–2.0). cDNA was synthesized from 1 µg of RNA using a reverse transcription kit with gDNA removal. The qPCR was conducted with SYBR Green pre-mix, following this protocol: initial denaturation at 95 °C for 3 minutes, 40 cycles of 95 °C for 10 seconds and 60 °C for 30 seconds, concluding with a melting curve analysis from 65 °C to 95 °C.The primer sequences for the target genes are as follows:

SIGLEC1: F 5′-CCAAGGTGGTGAAGTTCAAG-3′, R 5′-GCTCAGGACACAGTGGAGTG-3′

TRIM25: F 5′-GCTGGAGAAGGAGCTGATGA-3′, R 5′-TGGTGGTGTTGCTGTTGTAG-3′

Each sample included three technical replicates, and gene relative expression levels were determined using the 2^(−ΔΔCt) method, with GAPDH as the standard and the control group as the reference.

### SIGLEC1 knockdown

To investigate the functional role of SIGLEC1 in macrophage inflammatory responses, three independent shRNA constructs targeting SIGLEC1 (shSIGLEC1-1, shSIGLEC1-2, and shSIGLEC1-3) and a non-targeting negative-control construct (shNC) were introduced into THP-1-derived macrophages. Knockdown efficiency was evaluated by qPCR, and the construct showing the highest silencing efficiency was selected for subsequent functional experiments.

Enzyme-linked immunosorbent assay

For functional validation, THP-1-derived macrophages were divided into Control, LPS, LPS + shNC, and LPS + shSIGLEC1 groups. Following treatment, culture supernatants were collected, and IL-6 and TNF-α concentrations were measured using commercially available ELISA kits according to the manufacturer’s instructions. Absorbance was measured at 450 nm, and cytokine concentrations were calculated from standard curves.

### Flow cytometry

Macrophage activation phenotype was evaluated by flow cytometry using CD86 and CD206 staining. Following the indicated treatments, cells were harvested, washed, and stained with fluorochrome-conjugated antibodies according to the manufacturers’ instructions. Data were acquired using a flow cytometer and analyzed using FlowJo software.

## Results

### Differential screening

**Figure 2:**
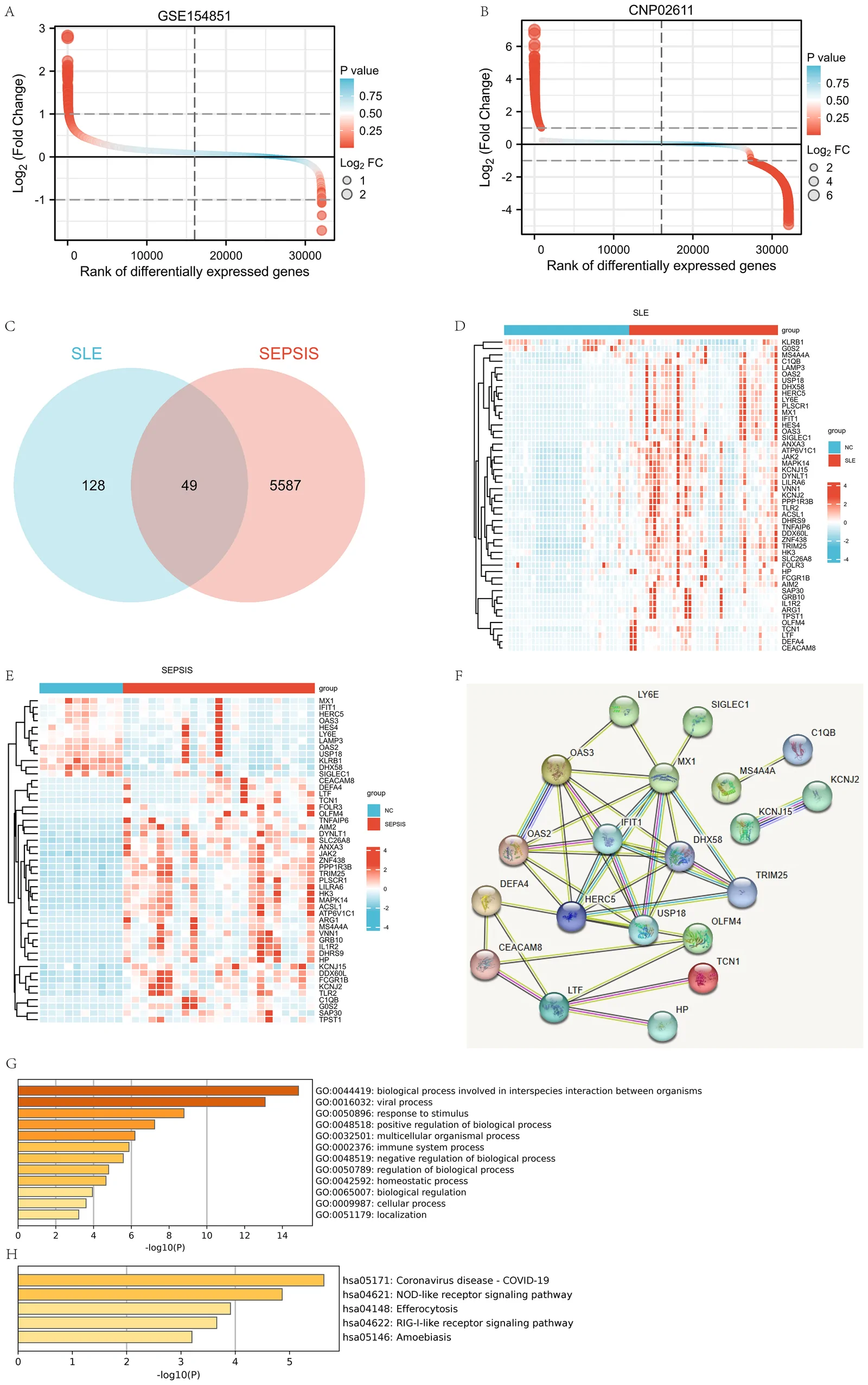
Identification and functional characterization of shared differentially expressed genes in SLE and sepsis. **(A, B)** Volcano plots showing differentially expressed genes in the GSE154851 SLE dataset and CNP02611 sepsis dataset, respectively. **(C)** Venn diagram showing the 49 shared candidate genes identified from the two discovery datasets. **(D, E)** Heatmaps showing the expression patterns of the shared candidate genes in GSE154851 and CNP02611, respectively. **(F)** STRING-derived protein–protein interaction network showing predicted interactions among the shared candidate genes. **(G)** Gene Ontology enrichment analysis of the shared candidate genes. **(H)** KEGG pathway enrichment analysis of the shared candidate genes.

**Figures 2A**, **B** display the volcano plots of differentially expressed genes (DEGs) identified in the GSE154851 and CNP02611 datasets, respectively. In volcano plots, gene significance is depicted by a color gradient from blue to red, reflecting decreasing P-values, while log2FoldChange indicates changes in gene expression, with dot size representing the extent of differential expression. The analysis revealed 128 significantly up-regulated genes in the GSE154851 dataset and 5,587 in the CNP02611 dataset. **Figure 2C** illustrates the intersection of significantly upregulated genes in the two diseases, with a total of 49 commonly upregulated genes screened. These genes could be significant in the development of SLE and Sepsis. **Figures 2D**, **E** show heatmaps of the expression levels of common differential genes in the SLE and Sepsis datasets, respectively. Red indicates gene up-regulation, while blue signifies gene down-regulation. **Figure 2D** illustrates the expression patterns of 49 significantly up-regulated common genes in the GSE154851 dataset, with **Figure 2E** confirming a similar trend in the CNP02611 dataset. The consistent expression patterns of the common genes across both datasets support further functional investigation of these genes.

Construction of the PPI network and subsequent enrichment analysis.

The STRING-derived PPI network contained 106 interactions among the shared candidate genes and provided an overview of their predicted protein-protein relationships (**Figure 2F**). PPI topology was used to characterize network structure and was not used as a standalone criterion for prioritizing SIGLEC1 or TRIM25.GO functional enrichment analysis revealed that the common differential genes were primarily associated with biological processes like interspecies interactions, viral processes, and responses to stimuli. These genes were also significantly linked to immune system processes and the regulation of biological processes, indicating their potential importance in immune regulation and disease defense (**Figure 2G**). KEGG pathway analysis indicated significant enrichment of these genes in the NOD-like receptor, RIG-I-like receptor, and coronavirus infection-associated pathways, implying their potential role in disease development via immune signaling and inflammatory response regulation (**Figure 2H**). To provide a transparent comparison across all 49 shared candidates, we performed an integrated prioritization analysis combining candidate-complete discovery-stage evidence with orthogonal supporting evidence (**Supplementary Table 1**). TRIM25 achieved an overall rank of 3/49, with concordant positive expression changes in both discovery datasets and a discovery-stage rank of 7/49. SIGLEC1 achieved an overall rank of 4/49; although its discovery-stage direction differed between the SLE and sepsis datasets, it received strong orthogonal support from single-cell localization, discriminatory performance, and protein-level evidence. SLC26A8 and PLSCR1 achieved slightly higher overall scores, whereas TRIM25 and SIGLEC1 were retained for downstream investigation as high-priority representative candidates because their complementary evidence profiles were particularly relevant to the cross-disease immune and single-cell focus of this study. Importantly, PPI connectivity was not included in the overall prioritization score.

### Immune infiltration analysis

**Figure 3:**
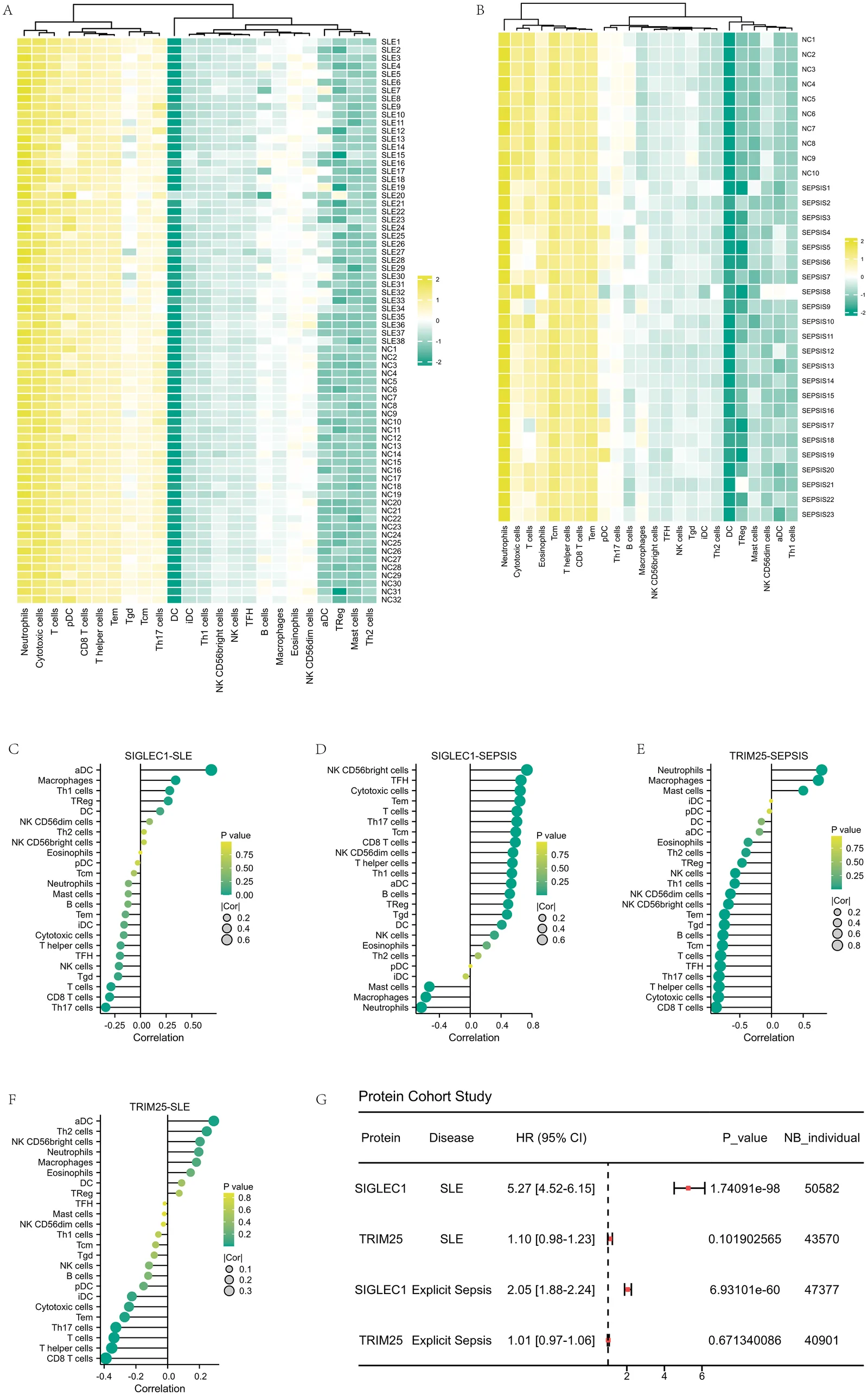
presents the outcomes of estimated immune-cell abundance analysis, the correlation between key genes and immune cells, and findings from protein cohort studies.Heatmaps **(A, B)** illustrate the enrichment score distribution for 28 immune cells in SLE **(A)** and Sepsis **(B)** samples.Yellow color indicates higher immune cell enrichment scores and blue-green color indicates lower scores. **(C–F)** Results of correlation analysis of key genes SIGLEC1 and TRIM25 with immune cell infiltration.Spearman correlation coefficients indicate correlations, with color denoting P-value magnitude and dot size reflecting correlation strength. **(C, D)** show the correlation of SIGLEC1 with immune cells in SLE and Sepsis, respectively, and **(E, F)** are the correlation of TRIM25 in Sepsis and SLE, respectively. **(G)** Results of protein cohort studies based on the Proteome-Phenome Atlas database demonstrating the association of plasma protein levels of SIGLEC1 and TRIM25 with SLE and Sepsis.Forest plots display hazard ratios (HR) and 95% confidence intervals, highlighting significant associations in red.

Immune infiltration analysis revealed notable differences in the infiltration patterns of various immune cells between SLE and Sepsis samples (**Figures 3A, B**). In the SLE samples, monocytes, dendritic cells (DCs), and T helper cells (Th1, Th2, etc.) exhibited notable enrichment scores. The Sepsis samples exhibited significantly higher enrichment scores for neutrophils and macrophages, indicating distinct cellular characteristics in the immune microenvironment of the two diseases. The correlation between two key genes, SIGLEC1 and TRIM25, and immune cell infiltration was further analyzed.SIGLEC1 showed a significant positive correlation with various immune cells in SLE, such as dendritic cells, monocytes, and macrophages (**Figure 3C**). In Sepsis samples, SIGLEC1 showed a significant positive correlation with macrophages, natural killer (NK) cells, and T helper cells (**Figure 3D**). TRIM25 exhibited a predominantly negative correlation with natural killer (NK) cells, monocytes, and B cells in SLE samples, while showing a significant positive correlation with neutrophils and macrophages in Sepsis samples. The findings indicate that SIGLEC1 and TRIM25 are associated with distinct immune-cell enrichment patterns in the two diseases, supporting their relationship with disease-related immune-cell states. Correlation coefficients between candidate genes and estimated monocyte/macrophage abundance scores are provided in **Supplementary Table 4**. Because SIGLEC1 showed predominant expression in myeloid populations according to single-cell analysis, we next evaluated whether its peripheral blood expression pattern was influenced by immune-cell composition changes. After adjustment for macrophage and neutrophil infiltration scores, the association between SIGLEC1 expression and disease status was substantially attenuated (β=-0.441, 95% CI: -2.843 to 1.962, P = 0.710), suggesting that the bulk transcriptomic signal of SIGLEC1 largely reflects alterations in myeloid-associated immune composition. In contrast, TRIM25 retained a modest association with disease status after immune-cell adjustment (β=0.446, 95% CI: 0.005–0.886, P = 0.047), indicating a partially independent disease-associated expression pattern (**Supplementary Figure 2**; **Supplementary Table 3**).

### Protein cohort study

The results showed that plasma protein levels of SIGLEC1 exhibited significant positive correlations with both systemic lupus erythematosus (SLE) and sepsis (Explicit Sepsis) based on analysis of the Proteome-Phenome Atlas database. In SLE, SIGLEC1 exhibited a risk ratio (HR) of 5.27, with a 95% confidence interval of [4.52-6.15] and a P value of 1.74×10^−98^, indicating its potential significance in SLE pathogenesis. In sepsis, SIGLEC1 exhibited a risk ratio of 2.05, with a 95% confidence interval of [1.88-2.24] and a P value of 6.93 × 10^−60^, indicating its potential involvement in immune-related diseases. Plasma protein levels of TRIM25 showed a hazard ratio (HR) of 1.10 (95% CI [0.98-1.23], P = 0.10) in SLE and 1.01 (95% CI [0.97-1.06], P = 0.67) in sepsis, with neither achieving statistical significance (**Figure 3G**). The findings indicate that SIGLEC1 protein-level associations provide additional evidence supporting its involvement as an immune-associated marker in SLE and sepsis, while TRIM25 appears less influential, offering a novel avenue for future research.

### Expression validation of key genes

**Figure 4:**
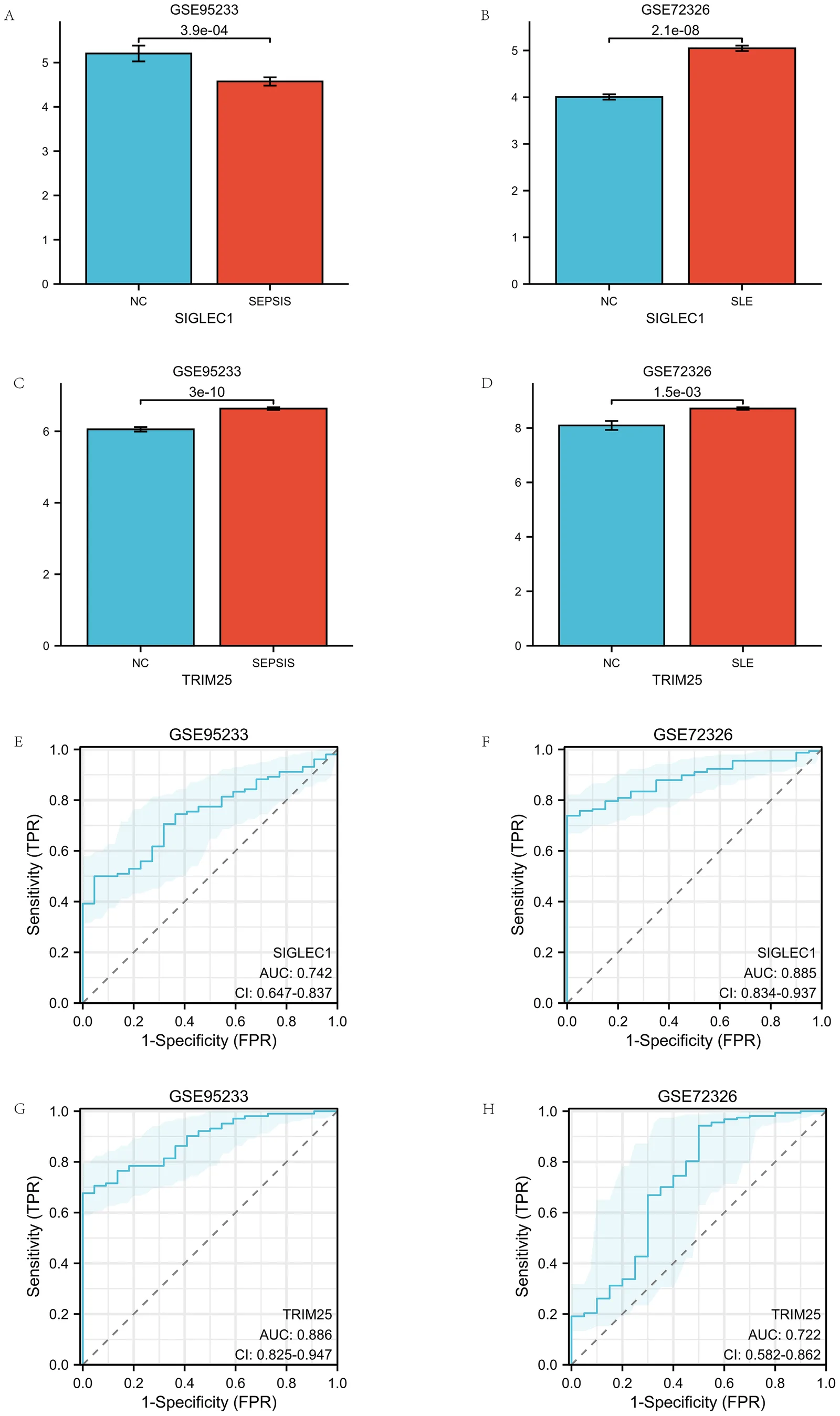
Expression levels and diagnostic efficacy analysis of key genes in sepsis and SLE. **(A, C)** Expression levels of SIGLEC1 and TRIM25 in sepsis patients (Sepsis) versus normal controls (NC) in the GSE95233 dataset. Significance is indicated by P-value, and bar graphs show mean ± standard error. Expression levels of SIGLEC1 and TRIM25 in systemic lupus erythematosus (SLE) patients compared to normal controls (NC) were analyzed using the GSE72326 dataset, with significance denoted by P values. **(E–H)** ROC curves assessing the diagnostic efficacy of SIGLEC1 and TRIM25 in Sepsis and SLE in the GSE95233 and GSE72326 datasets, with AUC (area under the curve) and 95% confidence intervals (CI) used to measure the diagnostic performance.

Differential expression analysis revealed significant changes in SIGLEC1 and TRIM25 expression in patients with both sepsis and systemic lupus erythematosus (SLE). In the GSE95233 dataset, sepsis patients exhibited significantly reduced SIGLEC1 expression compared to normal controls (P = 3.9 × 10^−4^, **Figure 4A**), whereas TRIM25 expression was significantly elevated (P = 3 × 10^−10^, **Figure 4C**). In the independent SLE validation cohort GSE72326, both SIGLEC1 and TRIM25 showed significantly increased expression in SLE patients compared with healthy controls (P = 1.6 × 10^−6^ and P = 5.8 × 10^−5^, respectively; **Figures 4B, D**). These findings indicate that SIGLEC1 and TRIM25, although identified as shared candidates during the discovery stage, may exhibit different expression patterns depending on disease context and cohort characteristics. The ROC curve analyses showed that SIGLEC1 and TRIM25 exhibited measurable discriminatory capacity between disease cohorts and healthy controls. In the GSE95233 dataset, the AUC of SIGLEC1 for Sepsis was 0.742 (95% CI: 0.647-0.837, **Figure 4E**), while that of TRIM25 was 0.886 (95% CI: 0.825-0.947, **Figure 4G**). In the GSE72326 dataset, the AUC of SIGLEC1 for SLE was 0.885 (95% CI: 0.834-0.937, **Figure 4F**), and the AUC of TRIM25 for SLE was 0.722 (95% CI: 0.582-0.862, **Figure 4H**). Given the relatively limited sample sizes of individual transcriptomic cohorts, the ROC analyses should be interpreted as exploratory assessments of discriminatory potential rather than definitive evidence of clinical diagnostic utility. These results suggest that SIGLEC1 and TRIM25 represent candidate immune-associated biomarkers with potential value for distinguishing disease cohorts from healthy controls. However, their clinical diagnostic utility requires further validation in clinically relevant differential-diagnosis cohorts. Although SIGLEC1 and TRIM25 were identified as shared upregulated genes in the initial discovery stage, independent validation revealed heterogeneous expression patterns across different cohorts. Specifically, SIGLEC1 showed reduced expression in the adult sepsis validation cohort but increased expression in SLE patients, whereas TRIM25 displayed heterogeneous expression patterns between individual validation cohorts and integrated meta-analysis results. These discrepancies may reflect differences in disease stage, cohort composition, immune status, and technical platforms. To further validate the expression patterns of SIGLEC1 and TRIM25 in systemic lupus erythematosus, we analyzed an independent whole-blood transcriptomic cohort (GSE72326). Both SIGLEC1 and TRIM25 showed significantly increased expression in SLE patients compared with healthy controls (**Supplementary Figure 1**).

### Meta-analysis

**Figure 5:**
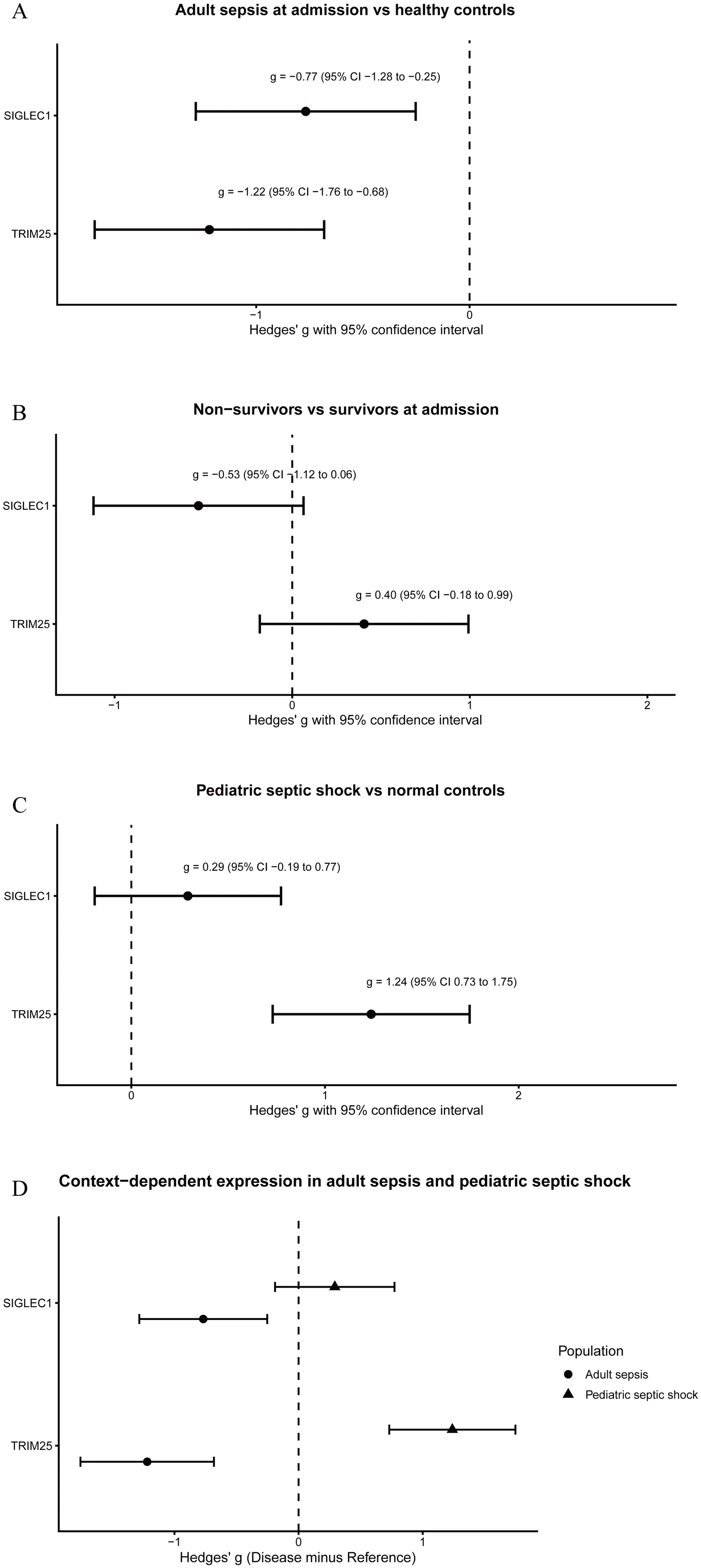
Cross-cohort effect-size comparison reveals context-dependent expression patterns of SIGLEC1 and TRIM25 in sepsis-related conditions. **(A)** Standardized effect sizes of SIGLEC1 and TRIM25 expression differences between adult sepsis patients and healthy controls. **(B)** Comparison of SIGLEC1 and TRIM25 expression between sepsis survivors and non-survivors. **(C)** Effect-size comparison between pediatric septic shock patients and normal controls. **(D)** Context-dependent expression patterns comparing adult sepsis and pediatric septic shock cohorts.

Utilizing independent sepsis-related transcriptomic cohorts, we performed cross-cohort effect-size analyses to evaluate whether the expression patterns of candidate genes TRIM25 and SIGLEC1 were consistent across different clinical contexts. These analyses revealed substantial heterogeneity in gene expression patterns across adult sepsis, pediatric septic shock, and outcome-associated cohorts. In adult sepsis cohorts.Detailed cohort-level expression changes and statistical parameters are summarized in **Supplementary Table 2**. TRIM25 exhibited a negative effect size compared with healthy controls (**Figure 5A**). However, this pattern was not consistently reproduced across different clinical settings, as pediatric septic shock cohorts showed an opposite direction of change (**Figure 5C**). These findings indicate that TRIM25 regulation may depend on patient age, disease stage, and immune status rather than representing a uniform alteration during sepsis. Although TRIM25 showed consistent directionality in some adult sepsis cohorts, the magnitude and direction of change varied among different clinical subgroups. Pediatric septic shock cohorts demonstrated an opposite expression pattern compared with adult sepsis cohorts, further highlighting the context-dependent regulation of TRIM25 during systemic inflammation. These findings suggest that TRIM25 reflects disease-associated immune states rather than a universal sepsis-specific expression signature.

In contrast, SIGLEC1 exhibited a heterogeneous expression profile across different clinical contexts. Although adult sepsis cohorts showed a negative effect direction compared with healthy controls, pediatric septic shock cohorts demonstrated a different pattern, indicating potential age- and disease-stage-dependent regulation of SIGLEC1. These findings suggest that SIGLEC1 regulation may depend on disease stage, patient characteristics, and immune-cell composition rather than representing a uniform change across sepsis. Interestingly, SIGLEC1 was significantly downregulated in pediatric sepsis cohorts, indicating possible age-dependent regulatory mechanisms. Furthermore, in disease models of SIRS and COVID-19, TRIM25 showed only mild suppression, whereas SIGLEC1 was markedly downregulated in COVID-19, potentially implicating an immunosuppressive role during viral infections.

Collectively, these cross-cohort analyses indicate that TRIM25 and SIGLEC1 exhibit context-dependent expression patterns across sepsis-related conditions. Rather than representing universal biomarkers with fixed expression directions, these genes may reflect disease-stage-specific and immune-context-dependent transcriptional states. Detailed cohort characteristics, comparison design, and effect-size information are summarized in **Supplementary Table 2**.

### Single-cell transcriptomic landscape of sepsis

**Figure 6:**
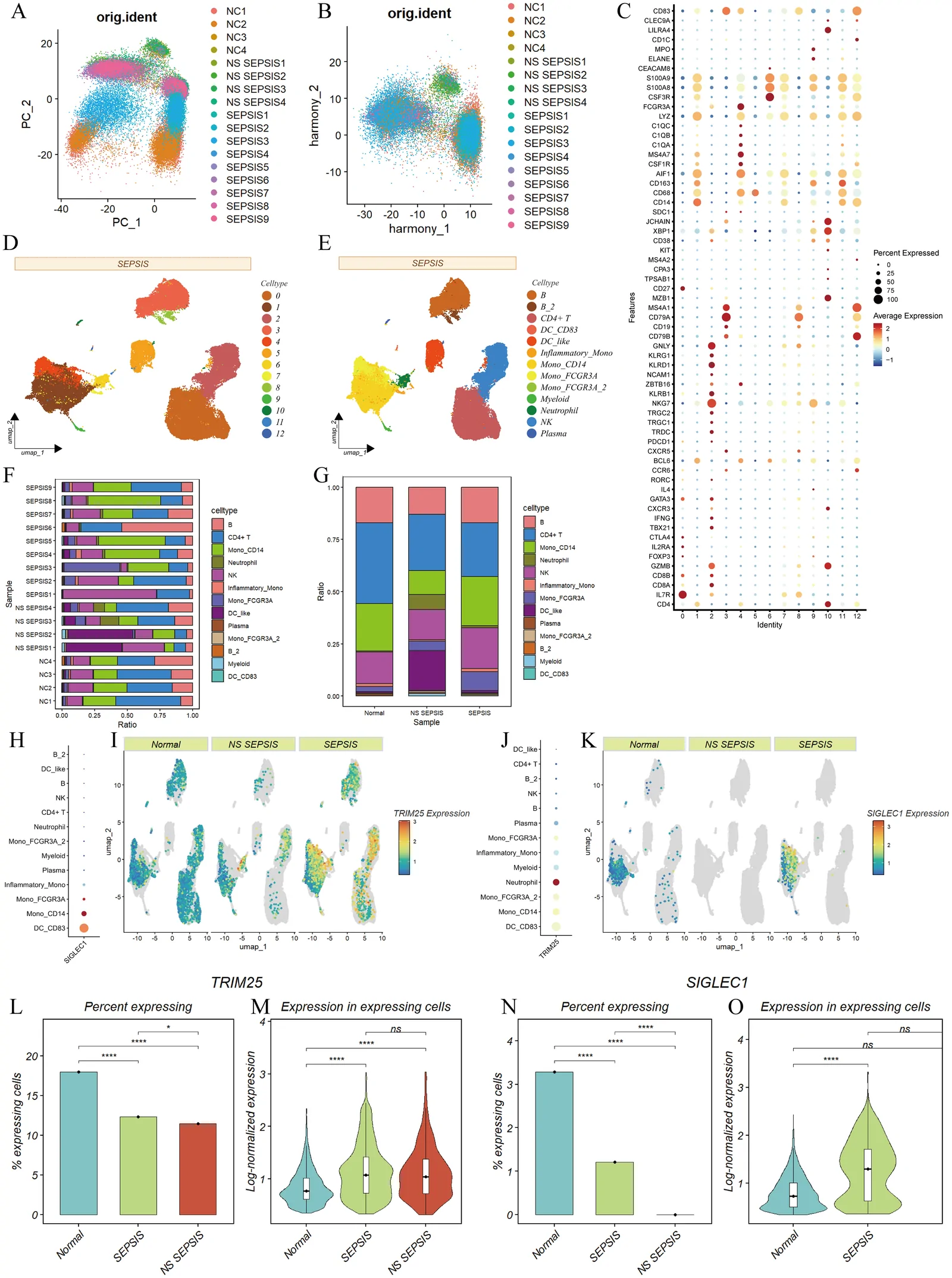
Analysis of sepsis samples using single-cell RNA sequencing, integrating both in-house and public datasets. Principal component analysis (PCA) plot prior to batch correction. UMAP visualization post-Harmony integration **(A)** PCA plot before batch correction. **(B)** UMAP plot. demonstrates successful elimination of batch effects across datasets. **(C–E)** UMAP plots illustrate cell clustering and annotation using canonical marker genes, identifying key immune cell populations such as T cells, B cells, NK cells, monocytes/macrophages, dendritic cells, and neutrophils. Images **(F, G)** present stacked bar plots illustrating the relative proportions of immune cell types across various samples and disease categories, including Normal, NS Sepsis, and Sepsis. **(H–J)** Feature plots illustrating TRIM25 expression across various immune cell types. Feature plots **(K–M)** illustrate the expression patterns of SIGLEC1 among various immune cell types. **(L–O)** Violin plots illustrating the differences in expression levels and the proportion of expressing cells for key genes among Normal, NS Sepsis, and Sepsis groups. ns indicates no significant difference; *P < 0.05; and ****P < 0.0001.

To comprehensively characterize the immune landscape of sepsis, we first analyzed five in-house scRNA-seq samples, including normal controls, SIRS, and sepsis patients, and further integrated these data with an independent public dataset (GSE167363) to enhance robustness. After quality control and batch correction using Harmony, cells were clustered into multiple immune populations and visualized using UMAP (**Figures 6A, B**). Using canonical markers for cell-type annotation, major immune cell subsets were identified: CD4^+^ T cells, CD8^+^ T cells, B cells, NK cells, monocytes/macrophages, dendritic cells, and neutrophils (**Figures 6C–E**). Notably, monocytes and macrophages constituted a dominant fraction in sepsis samples, whereas lymphocyte populations were relatively reduced (**Figures 6F, G**), indicating a shift toward innate immune activation. Subsequently, we analyzed the expression patterns of essential genes. TRIM25 was widely expressed in various immune cells, such as T cells, monocytes, and NK cells, indicating its overarching role in immune regulation (**Figures 6H–J**).In contrast, SIGLEC1 showed a more restricted expression pattern, being predominantly enriched in monocyte/macrophage populations (**Figures 6K–M**). Quantitative analysis revealed notable differences in the proportion of expressing cells and expression levels across Normal, NS Sepsis, and Sepsis groups (**Figures 6L–O**), underscoring the disease-related activation of these genes. However, because cell-level differential expression may be affected by unequal numbers of cells derived from different individuals, we further performed patient-level pseudobulk analysis. In this analysis, each sample rather than each cell was considered as the statistical unit. The pseudobulk results confirmed the cell-type-associated expression pattern of SIGLEC1 and TRIM25, while also revealing disease- and cell-context-dependent expression differences (**Supplementary Figure 3**).

### Cross-disease validation in SLE single-cell datasets

**Figure 7:**
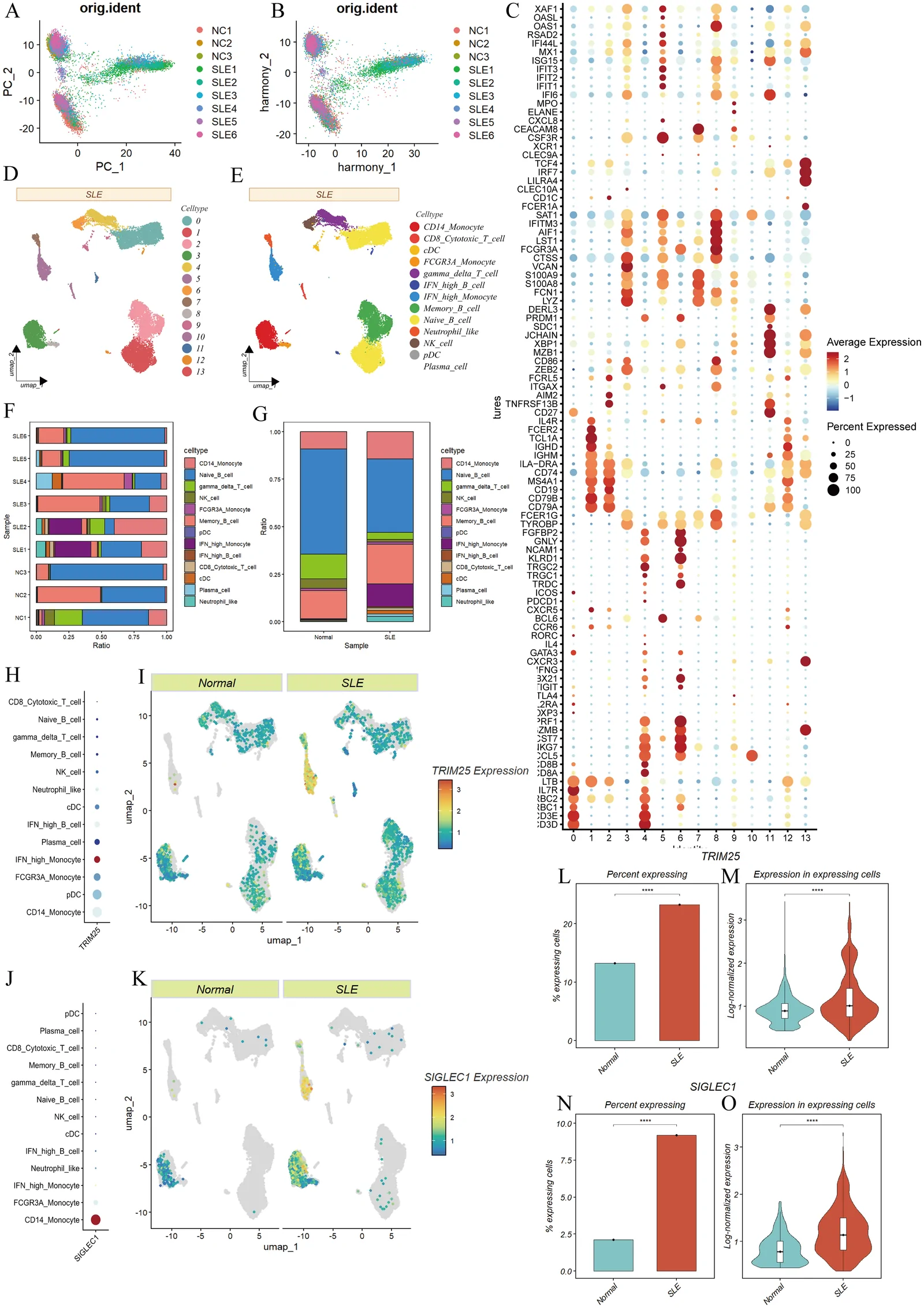
Analysis of single-cell RNA sequencing data from independent public datasets for systemic lupus erythematosus (SLE). UMAP plots **(A, B)** display integrated single-cell clustering and cell-type annotation using canonical marker genes. Visualization of key immune cell populations, such as T cells, B cells, monocytes/macrophages, dendritic cells, and NK cells. **(F, G)** Stacked bar plots showing the distribution of immune cell types across Normal and SLE groups. **(H, J)** Feature plots illustrating TRIM25 expression across various immune cell subsets. Feature plots **(K–M)** illustrate SIGLEC1 expression patterns across various immune cell subsets. **(L–O)** Violin plots showing the differential expression levels and proportion of expressing cells for key genes between the Normal and SLE groups. ****P < 0.0001.

To determine whether these findings are conserved in autoimmune conditions, we further analyzed scRNA-seq data from SLE cohorts (GSE162577 and GSE136035). Post-integration and clustering, cells were categorized into immune populations akin to those in sepsis, such as T cells, B cells, monocytes, dendritic cells, and NK cells (**Figures 7A–E**). Compared with sepsis, SLE samples displayed a distinct immune composition characterized by a higher proportion of adaptive immune cells, particularly T and B lymphocytes (**Figures 7F, G**), consistent with the autoimmune nature of the disease. Expression analysis revealed that TRIM25 remained broadly expressed across multiple immune cell populations, with prominent enrichment in T cell subsets (**Figures 7H–J**). Meanwhile, SIGLEC1 was still preferentially expressed in monocyte/macrophage populations, although its overall expression level was elevated in SLE compared with normal controls (**Figures 7K–M**). Statistical analysis revealed notable increases in both the proportion of expressing cells and the expression intensity in disease samples (**Figures 7L–O**). These findings indicate that although the cellular localization of key genes is conserved across diseases, the dominant immune context differs, with innate immune activation in sepsis and adaptive immune predominance in SLE.

### Functional characterization by pathway activity analysis

**Figure 8:**
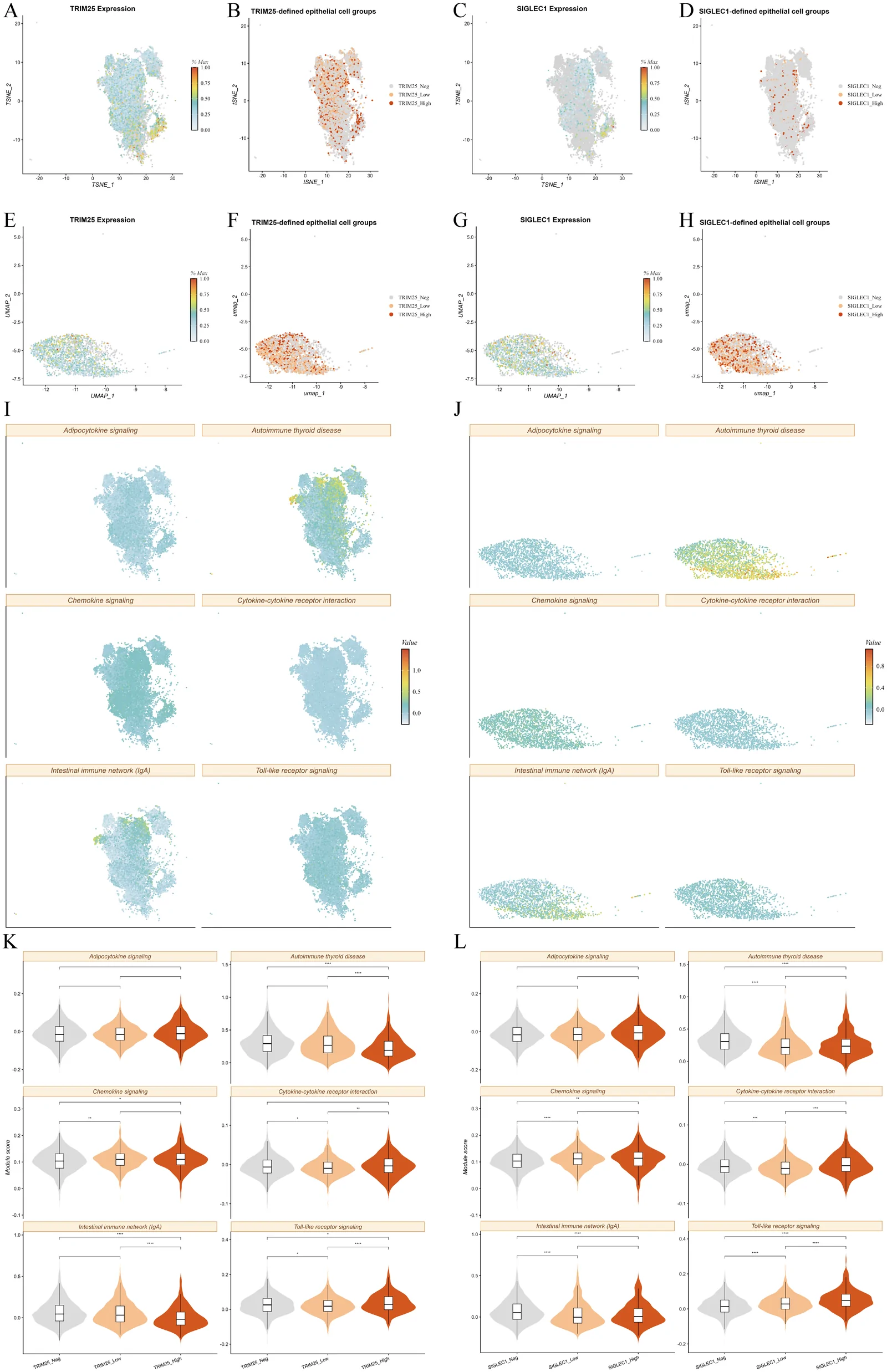
Analysis of immune pathway activity in individual cells for sepsis and SLE. **(A–D)** Heatmaps showing pathway activity scores calculated by GSVA across different immune cell populations. Comparative analysis of immune-related pathways, such as interferon signaling, cytokine–cytokine receptor interaction, Toll-like receptor signaling, and antigen processing and presentation, across various disease conditions. **(I–L)** Analysis of pathway activity stratified by gene expression levels, highlighting differences between high and low expression groups for TRIM25 and SIGLEC1. Overall, these analyses highlight both shared and disease-specific immune regulatory pathways in sepsis and SLE. **(E–H)** UMAP plots showing TRIM25 expression/groups and SIGLEC1 expression/groups. *P < 0.05; **P < 0.01; ***P < 0.001; and ****P < 0.0001.

To further investigate the functional implications of these cellular and transcriptional differences, pathway activity scoring was performed at the single-cell level using GSVA (**Figure 8**). Sepsis and SLE samples showed enrichment in immune-related pathways, such as cytokine–cytokine receptor interaction, Toll-like receptor signaling, and interferon-related responses. However, distinct patterns were observed between the two diseases. In sepsis, pathway activity was predominantly enriched in innate immune and inflammatory signaling, particularly within monocyte/macrophage populations.SLE samples exhibited enhanced activation of adaptive immune pathways, such as antigen processing and presentation, T cell receptor signaling, and B cell responses. Furthermore, stratification based on gene expression levels revealed that cells with high TRIM25 or SIGLEC1 expression were associated with enhanced immune pathway activation, suggesting that these genes may reflect disease-associated immune transcriptional states. These findings indicate that although sepsis and SLE share inflammatory signaling pathways, their primary immune programs differ significantly, highlighting the distinct roles of key genes in each disease context.

### Key gene expression validation

**Figure 9:**
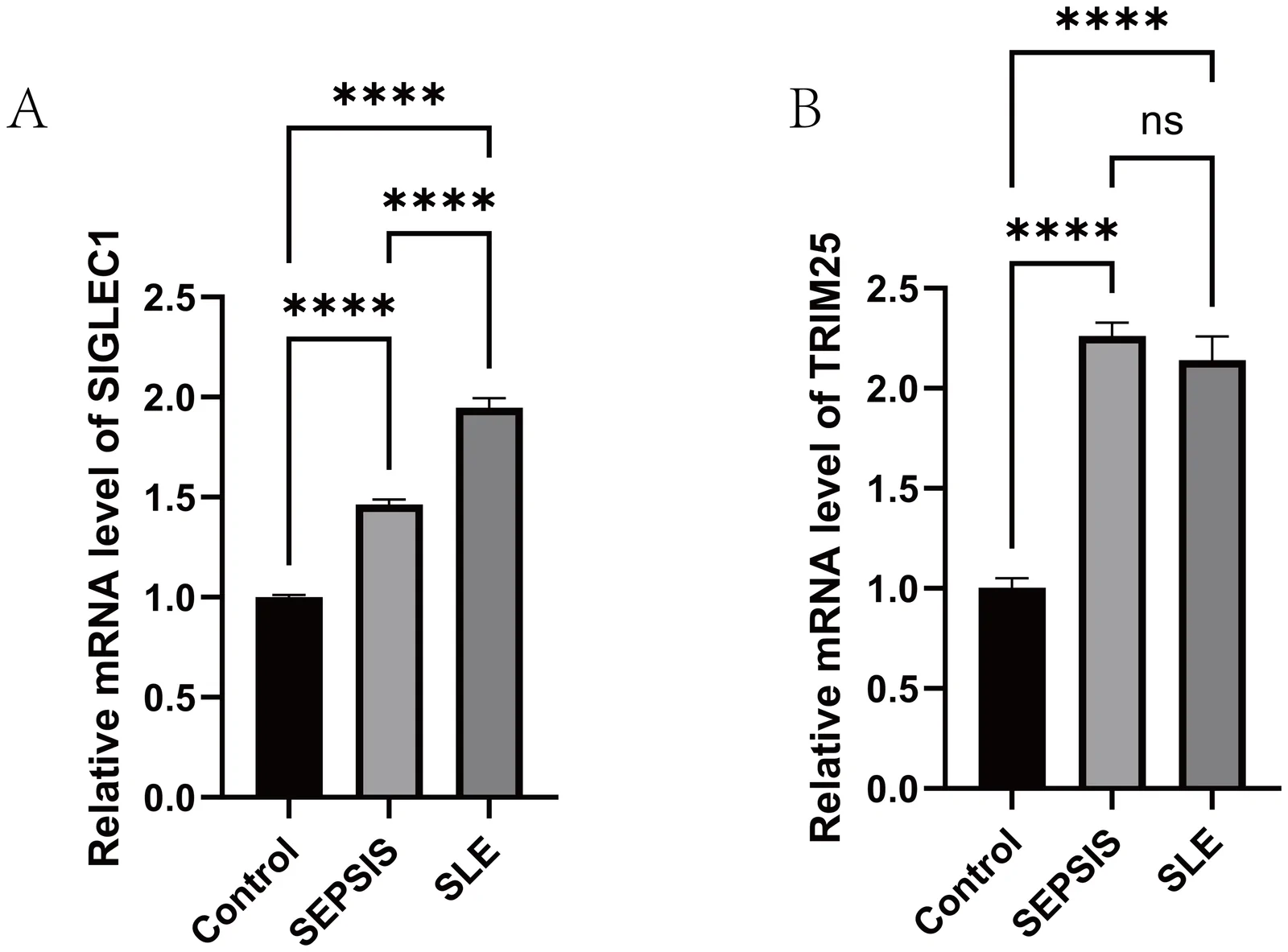
Relative mRNA levels of key genes in different THP - 1 macrophage models. Quantitative PCR analysis of SIGLEC1 mRNA expression levels. **(B)** Relative mRNA level of TRIM25 detected by qPCR.Data are expressed as mean ± SEM.**** denotes P < 0.0001, while ns signifies no significant difference.

After inducing THP-1 monocyte - to - macrophage differentiation and establishing *in vitro* inflammatory and immune-metabolic macrophage models (control, LPS-induced inflammatory activation, and 4-OI-induced itaconate-associated immunometabolic modulation), we detected the relative mRNA levels of SIGLEC1 and TRIM25 via qPCR.For SIGLEC1 (**Figure 9A**), its expression was significantly up-regulated in both the sepsis and SLE groups compared to the control group (P < 0.0001). Moreover, the SLE group showed a higher expression level than the sepsis group (P < 0.0001). Regarding TRIM25 (**Figure 9B**). The sepsis group showed a significant elevation in mRNA levels compared to the control group (****, P < 0.0001). TRIM25 expression levels were elevated in the SLE group compared to the control, but no significant difference was observed between the SLE and sepsis groups.

### Functional validation of SIGLEC1 in LPS-stimulated macrophages

**Figure 10:**
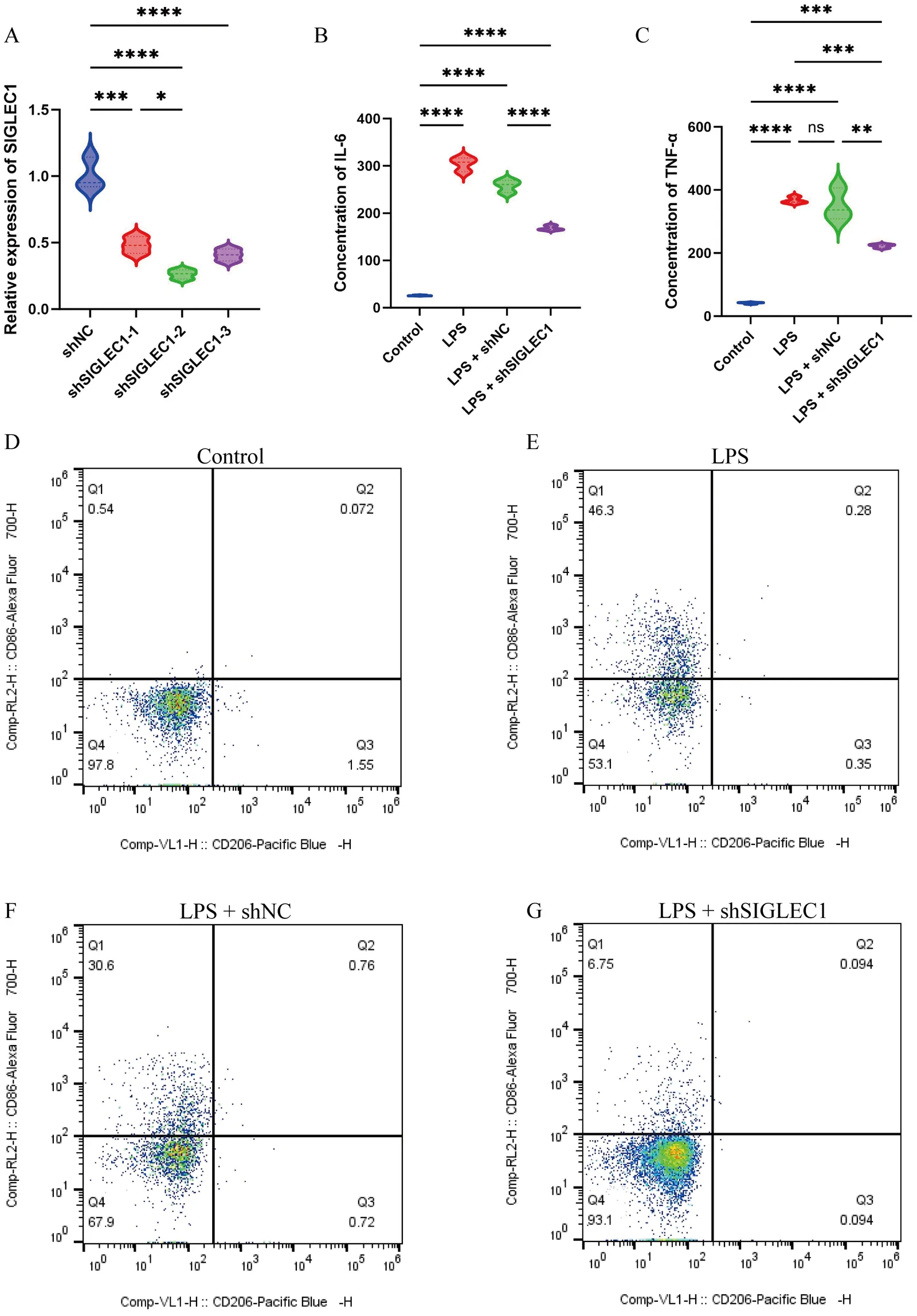
Functional validation of SIGLEC1 in LPS-stimulated THP-1-derived macrophages. **(A)** qPCR screening of three independent shRNAs targeting SIGLEC1; shSIGLEC1–2 showed the greatest knockdown efficiency and was selected for subsequent experiments. **(B, C)** ELISA analysis of IL-6 and TNF-α concentrations in culture supernatants from Control, LPS, LPS + shNC, and LPS + shSIGLEC1 groups. **(D–G)** Representative flow cytometry plots showing CD86 and CD206 expression patterns in Control, LPS, LPS + shNC, and LPS + shSIGLEC1 groups, respectively. Data are presented as mean ± SEM. *P < 0.05, **P < 0.01, ***P < 0.001, ****P < 0.0001; ns, not significant.

To further determine whether SIGLEC1 functionally contributes to macrophage inflammatory responses, three independent shRNA constructs targeting SIGLEC1 were first screened. qPCR analysis showed that shSIGLEC1–2 produced the strongest reduction in SIGLEC1 expression and was therefore selected for subsequent functional experiments (**Figure 10A**). LPS stimulation markedly increased IL-6 and TNF-α secretion compared with the control group. SIGLEC1 knockdown significantly reduced the LPS-induced production of both cytokines compared with the LPS + shNC group (**Figures 10B, C**). Flow cytometric analysis further showed that LPS stimulation markedly increased the CD86-positive macrophage population, whereas SIGLEC1 knockdown attenuated this LPS-induced CD86-positive phenotype compared with the LPS + shNC group (**Figures 10D–G**). Collectively, these findings indicate that SIGLEC1 contributes to LPS-induced inflammatory activation of THP-1-derived macrophages.

## Discussion

In this study, by integrating bulk transcriptomic, single-cell, cross-cohort, and experimental data, we identified SIGLEC1 and TRIM25 as high-priority immune-associated candidates in systemic lupus erythematosus (SLE) and sepsis and characterized their context-dependent expression and cellular distribution patterns.

SIGLEC1, also referred to as CD169 or sialoadhesin, is crucial in modulating immune responses and inflammation within the immune system. SIGLEC1 is an adhesion molecule primarily located on the surface of human myeloid cells. SIGLEC1, part of the salivary acid-binding immunoglobulin-like lectin family, binds to acidified molecular structures in various ligands. It shows promise as a diagnostic biomarker for distinguishing between viral and bacterial infections in both respiratory and non-respiratory contexts, addressing a persistent challenge in infectious disease management. an ongoing challenge (28, 29).

In COVID-19 patients, the expression level of SIGLEC1 correlated with disease severity. It was found that the expression level of CD169/SIGLEC1 on monocytes was higher in patients with mild acute COVID-19 and lower in patients with severe disease. This indicates that SIGLEC1 expression could be linked to the immune response in COVID-19 and might act as a potential marker for evaluating the condition of COVID-19 patients (30). In addition, SIGLEC1 is present as an active neuroinflammatory marker in the brains of multiple sclerosis (MS) patients, but is not evident in the blood. Research indicates that SIGLEC1+ myeloid cells are prevalent in both acute and chronic MS brain injuries, implying a potential role for SIGLEC1 in central nervous system inflammation (31). In inflammatory diseases, the regulation of SIGLEC1 function and expression may influence disease progression and immune responses. In ankylosing spondylitis (AS), reducing SIGLEC1 expression can impede disease progression by modulating the TGF-β1/SMAD signaling pathway, thereby enhancing cell proliferation and reducing osteogenic differentiation and inflammation (32). These studies revealed multiple roles of SIGLEC1 in immunomodulation and inflammation and provided new perspectives for future therapeutic strategies. This study identified SIGLEC1 as an immune-related candidate shared between SLE and sepsis, particularly in monocyte and macrophage populations. Although SIGLEC1 showed increased expression in SLE validation cohorts, its expression pattern varied across sepsis cohorts, suggesting that its immunological role may depend on disease context. Immune infiltration analysis indicated that elevated SIGLEC1 expression is strongly linked to increased monocyte and macrophage presence, underscoring its significant role in the immune microenvironment. ROC analysis demonstrated that SIGLEC1 exhibited discriminatory capacity between disease cohorts and healthy controls. However, these findings should be interpreted as exploratory evidence rather than clinical diagnostic validation.

Importantly, the present study extended these association-based findings through loss-of-function validation. Silencing SIGLEC1 in LPS-stimulated THP-1-derived macrophages reduced IL-6 and TNF-α secretion and attenuated the CD86-positive macrophage phenotype. These findings provide initial functional evidence that SIGLEC1 contributes to macrophage inflammatory activation under LPS stimulation. However, the downstream molecular mechanisms remain unresolved, and the present experiments do not establish a complete causal pathway.

TRIM25 is a crucial E3 ubiquitin ligase involved in various cellular functions, such as immune response and inflammation regulation. TRIM25 is crucial in innate immunity by facilitating RIG-I ubiquitination and boosting its antiviral signaling (33, 34). TRIM25 regulates inflammatory responses by interacting with proteins that control various cell signaling pathways (35). During inflammation, TRIM25 affects intercellular communication by selectively loading miRNAs into extracellular vesicles. The TRIM25-mediated K63-ubiquitination of FMR1 is crucial for its interaction with the vesicle loading machinery (36). In addition, TRIM25 inhibits viral replication by targeting specific proteins for ubiquitination and degradation. TRIM25 suppresses hepatitis B virus replication by facilitating HBx degradation (37).TRIM25 is crucial in the development and progression of both inflammatory diseases and cancer. The regulation of TRIM25 expression is linked to the progression of various diseases, making it a potential disease-associated molecule for further mechanistic investigation (38). In some cases, over-activation of TRIM25 may lead to excessive inflammatory responses, thereby exacerbating the severity of the disease (39). Taken together, TRIM25 has multiple functions in immune and inflammatory responses, influencing the immune response and inflammatory state of cells by regulating signaling pathways and protein interactions. These studies offer insights into TRIM25’s role in disease mechanisms and suggest potential therapeutic strategies. This study identified TRIM25 as an immune-related candidate involved in both SLE and sepsis. Although increased expression was observed in the initial discovery datasets and SLE validation cohort, subsequent analyses revealed heterogeneous regulation across sepsis cohorts, indicating that TRIM25 may participate in disease-specific immune responses rather than displaying a universal expression pattern. In addition, ROC analysis indicated that TRIM25 showed potential discriminatory capacity in sepsis cohorts compared with healthy controls. Further studies involving clinically relevant control populations are required to determine whether TRIM25 can provide additional diagnostic value in real-world clinical settings.

However, the biological functions of SIGLEC1 and TRIM25 are not exclusively restricted to promoting inflammatory activation. Increasing evidence indicates that the effects of these molecules may depend on cellular context, inflammatory stimuli, and disease stage. SIGLEC1 expression reflects myeloid-cell activation and has been associated with inflammatory states in multiple diseases; however, its role may also involve immune regulation and modulation of cell-cell interactions under specific conditions. Likewise, TRIM25 participates in innate immune signaling through regulation of antiviral and inflammatory pathways, but its downstream effects may vary according to the specific signaling environment and feedback mechanisms. Therefore, the differential expression patterns of SIGLEC1 and TRIM25 observed across sepsis and SLE cohorts may represent disease-specific immune adaptations rather than simple indicators of enhanced inflammation. These findings highlight the importance of interpreting immune regulatory molecules within their biological context.

Importantly, the differences observed among datasets do not necessarily represent contradictory findings. Variations in disease mechanisms, clinical stages, immune-cell composition, patient characteristics, and transcriptomic platforms may contribute to heterogeneous gene expression patterns. Therefore, SIGLEC1 and TRIM25 should be interpreted as context-dependent immune-associated candidates rather than universal biomarkers with identical expression changes across all inflammatory diseases.

Compared with previous studies on SLE and Sepsis, which mainly focused on exploring the molecular mechanism of a single disease or association analysis based on a small number of genes. Our findings suggest that the shared biological basis between sepsis and SLE does not represent a simple overlap of generalized inflammatory responses. Instead, these diseases appear to converge on partially shared immune regulatory programs, including myeloid-cell-associated regulation, interferon-related signaling, and innate immune activation. SIGLEC1 mainly reflects monocyte/macrophage-associated immune regulation, whereas TRIM25 represents an interferon-related regulatory component with broader immune-cell distribution. This integrated analysis at the gene level revealed shared immune-associated programs between SLE and sepsis and identified SIGLEC1 and TRIM25 as candidate biomarkers with context-dependent expression patterns. These findings provide a foundation for future mechanistic studies to determine their functional roles and therapeutic relevance.

The integrated prioritization analysis further demonstrated that candidate selection could not be reduced to a single ranking metric. Although SLC26A8 and PLSCR1 achieved the two highest overall scores, TRIM25 and SIGLEC1 ranked third and fourth, respectively, and showed complementary evidence profiles that were particularly relevant to the biological questions addressed in this study. TRIM25 was characterized by concordant discovery-stage expression and additional cross-cohort and single-cell support, whereas SIGLEC1 was distinguished by strong myeloid-cell localization, discriminatory performance, and protein-level evidence. Therefore, their selection was based on high integrated prioritization together with biological complementarity rather than PPI connectivity or top-two ranking alone.

In addition, this study used a proteome database (Proteome-Phenome Atlas) to validate the protein levels of key genes with disease correlations. This strategy compensates for the limitation of relying only on gene expression data and provides more comprehensive evidence for the clinical translation of SIGLEC1 and TRIM25 in SLE and Sepsis. The advent of single-cell sequencing data has highlighted the cell-type specificity of key genes, providing a basis for investigating their roles in the disease microenvironment.

Previous studies have demonstrated that the ACOD1/itaconate pathway is involved in immune regulation during autoimmune inflammation, including systemic lupus erythematosus. In particular, pharmacological modulation of itaconate signaling by 4-octyl itaconate has been shown to influence lupus-associated inflammatory responses in experimental models. Therefore, 4-OI treatment was selected in this study as an approach to investigate itaconate-associated immunometabolic regulation related to SLE pathophysiology rather than as a complete *in vitro* SLE disease model. Nevertheless, THP-1-derived macrophage systems cannot fully reproduce the complexity of SLE, and future validation using patient-derived monocytes/PBMCs, interferon-α stimulation, immune-complex stimulation, or plasma-transfer models will be necessary.

Although this study validated the important roles of SIGLEC1 and TRIM25 in SLE and Sepsis through multiple approaches, several limitations remain. Although data were sourced from various public databases, the study sample’s population diversity and size may still be inadequate to fully capture the heterogeneity of the two diseases. Future studies should include more races and larger samples to improve the generalizability of the results. Although this study incorporated SIGLEC1 loss-of-function experiments in THP-1-derived macrophages, these experiments provide only initial functional evidence and do not establish a complete causal mechanism linking SIGLEC1 to disease progression. Further rescue experiments, gain-of-function studies, primary patient-derived immune-cell validation, and *in vivo* experiments will be required to define the downstream mechanism and disease relevance of SIGLEC1. In addition, the functional contribution of TRIM25 remains to be experimentally investigated.

## Data Availability

The datasets presented in this study can be found in online repositories. The names of the repository/repositories and accession number(s) can be found in the article/**Supplementary Material**.

## Supplementary material

Supplementary Figure 1 Expression validation of SIGLEC1 and TRIM25 in an independent systemic lupus erythematosus cohort. **(A)** Expression comparison of SIGLEC1 between healthy controls and SLE patients from the GSE72326 whole-blood transcriptomic dataset. **(B)** Expression comparison of TRIM25 between healthy controls and SLE patients from the GSE72326 cohort. Statistical significance was assessed using the Wilcoxon rank-sum test. P values are indicated in the plots.

Supplementary Figure 2 Association between SIGLEC1/TRIM25 expression and immune-cell composition and cell-composition-adjusted regression analysis. **(A, B)** Correlation between SIGLEC1 expression and estimated monocyte/macrophage abundance scores in peripheral blood samples. **(C, D)** Correlation between TRIM25 expression and estimated monocyte/macrophage abundance scores. **(E, F)** Unadjusted and cell-composition-adjusted regression models evaluating the association between disease status and SIGLEC1/TRIM25 expression. Adjusted models included macrophage and neutrophil scores as covariates. Beta values represent the estimated difference in log2(expression + 1) between sepsis and control groups.

Supplementary Figure 3 Patient-level pseudobulk validation of SIGLEC1 and TRIM25 expression patterns in single-cell transcriptomic datasets. **(A, B)** Summary of pseudobulk differential expression analysis in SLE and sepsis datasets using individual samples as statistical units. **(C, D)** Patient-level expression distributions of SIGLEC1 and TRIM25 in SLE CD14+ monocytes. **(E, F)** Patient-level expression distributions of SIGLEC1 and TRIM25 in sepsis CD14+ monocytes. **(G, H)** Patient-level expression distributions of SIGLEC1 and TRIM25 in sepsis neutrophils. Each dot represents an individual patient/sample. Differential expression analysis was performed using pseudobulk aggregation followed by count-based statistical testing.
