# Supplementary material for: Single-cell profiling reveals context-dependent immune regulation shared by sepsis and systemic lupus erythematosus
Source: Front Immunol. 2026 Sep 3;17:1892194. doi: 10.3389/fimmu.2026.1892194 (PMC13581862; doi:10.3389/fimmu.2026.1892194)
Supplement: Supplementary file 1 [file DataSheet1.pdf]

frontiers

The International Society for the Advancement of Cytometry (ISAC) has highlighted the importance of including comprehensive methodological information to ensure data reproducibility and reliability. In line with this, Frontiers in Immunology now requires authors to submit a checklist for manuscripts that involve flow or mass cytometry. This checklist helps standardize the reporting process, improving the quality and transparency of published data. By doing so, we support scientific progress, making it easier for other researchers to replicate and validate experiments.

This form should be submitted with any manuscripts using flow or mass cytometry.

Sample/specimen/material description

☐ Total blood

☐ PBMCs

☐ Organ digests

Other PMA-differentiated THP-1-derived macrophages

Did the samples suffer any treatment before or after incubation with the antibodies?

☐ Drug ____

☐ Cell permeabilization ____

☐ Dye ____

☐ Propidium iodine

☐ Not applicable

Other PMA differentiation followed by LPS stimulation and/or SIGLEC1 shRNA transduction according to the experimental group

Instrument and antibodies

Name of the Cytometer BD FACSCanto II

Antibodies and targets	Fluorochrome/ Metal	Catalog number/Company
anti-human CD86	PerCP/Cyanine5.5	Cat. 374216 / BioLegend

anti-human CD206	Pacific Blue	Cat. 321152 / BioLegend

Data analyses

1. Name of the software FlowJo v10.2 ______________________________
2. Reference gating strategy in the manuscript or supplementary material

Gating strategy in (eg Figure X) Supplementary Material: Flow Cytometry Gating Strategy ______________________________
