# Supplementary material for: Single-cell profiling reveals context-dependent immune regulation shared by sepsis and systemic lupus erythematosus
Source: Front Immunol. 2026 Sep 3;17:1892194. doi: 10.3389/fimmu.2026.1892194 (PMC13581862; doi:10.3389/fimmu.2026.1892194)
Supplement: Supplementary file 2 [file Table1.docx]

Supplementary Table S1. Integrated comparative prioritization of the 49 shared immune-associated candidate genes.

| **Overall rank** | **Gene** | **SLE log2FC** | **Sepsis log2FC** | **Direction** | **Discovery rank** | **Orthogonal score** | **Overall score** | **Supporting evidence code** |
| --- | --- | --- | --- | --- | --- | --- | --- | --- |
| 1 | SLC26A8 | 1.053 | 2.205 | Concordant positive | 4 | 0.790 | 74.12 | P;S;Pr |
| 2 | PLSCR1 | 1.629 | 1.942 | Concordant positive | 1 | 0.636 | 71.92 | P;V;Pr |
| 3 | **TRIM25** | 1.230 | 1.163 | Concordant positive | 7 | 0.712 | 69.99 | V;S |
| 4 | **SIGLEC1** | 1.607 | -2.510 | Discordant | 12 | 0.734 | 67.19 | S;R;Pr |
| 5 | ANXA3 | 1.211 | 4.312 | Concordant positive | 3 | 0.509 | 62.41 | V;R |
| 6 | G0S2 | -1.716 | 3.660 | Discordant | 9 | 0.541 | 61.19 | P;S;R |
| 7 | GRB10 | 1.393 | 2.982 | Concordant positive | 4 | 0.524 | 60.81 | P;V |
| 8 | DDX60L | 1.448 | 1.108 | Concordant positive | 10 | 0.571 | 59.55 | V |
| 9 | AIM2 | 1.558 | 1.764 | Concordant positive | 7 | 0.473 | 58.00 | — |
| 10 | FCGR1B | 1.045 | 2.361 | Concordant positive | 16 | 0.561 | 56.71 | V;R |
| 11 | MS4A4A | 1.093 | 2.653 | Concordant positive | 19 | 0.570 | 56.65 | P;V;R |
| 12 | HP | 1.556 | 4.964 | Concordant positive | 2 | 0.382 | 56.58 | P;R |
| 13 | TNFAIP6 | 1.092 | 2.884 | Concordant positive | 15 | 0.536 | 55.71 | S |
| 14 | KCNJ2 | 1.541 | 1.048 | Concordant positive | 25 | 0.595 | 55.28 | V;R |
| 15 | USP18 | 1.880 | -1.939 | Discordant | 12 | 0.466 | 53.78 | R |
| 16 | MAPK14 | 1.190 | 1.842 | Concordant positive | 4 | 0.381 | 53.69 | P |
| 17 | LILRA6 | 1.042 | 1.962 | Concordant positive | 21 | 0.526 | 53.64 | — |
| 18 | OLFM4 | 1.381 | 4.289 | Concordant positive | 22 | 0.518 | 53.00 | — |
| 19 | DYNLT1 | 1.213 | 1.372 | Concordant positive | 19 | 0.487 | 52.46 | — |
| 20 | OAS2 | 1.546 | -1.801 | Discordant | 16 | 0.454 | 51.37 | V |
| 21 | LTF | 1.073 | 3.254 | Concordant positive | 43 | 0.682 | 50.75 | V |
| 22 | CEACAM8 | 1.091 | 3.384 | Concordant positive | 36 | 0.615 | 50.52 | P;R;Pr |
| 23 | HK3 | 1.186 | 2.419 | Concordant positive | 14 | 0.401 | 49.23 | R |
| 24 | VNN1 | 1.104 | 3.714 | Concordant positive | 11 | 0.370 | 49.22 | R |
| 25 | IFIT1 | 1.940 | -2.272 | Discordant | 24 | 0.442 | 47.90 | R |
| 26 | DHX58 | 1.168 | -1.360 | Discordant | 32 | 0.527 | 47.71 | V |
| 27 | KLRB1 | -1.077 | -2.713 | Concordant negative | 30 | 0.503 | 47.00 | P;R |
| 28 | HERC5 | 1.608 | -1.408 | Discordant | 36 | 0.544 | 46.99 | V |
| 29 | TCN1 | 1.005 | 2.939 | Concordant positive | 38 | 0.530 | 46.05 | V;R |
| 30 | IL1R2 | 1.068 | 2.644 | Concordant positive | 35 | 0.517 | 45.89 | V |
| 31 | ARG1 | 1.142 | 4.437 | Concordant positive | 16 | 0.344 | 45.83 | — |
| 32 | LAMP3 | 1.182 | -1.525 | Discordant | 40 | 0.547 | 45.60 | R |
| 33 | OAS3 | 1.310 | -1.591 | Discordant | 27 | 0.431 | 45.23 | V |
| 34 | ZNF438 | 1.032 | 1.740 | Concordant positive | 28 | 0.420 | 43.89 | — |
| 35 | TLR2 | 1.114 | 1.148 | Concordant positive | 38 | 0.480 | 43.55 | — |
| 36 | JAK2 | 1.008 | 1.244 | Concordant positive | 23 | 0.318 | 42.20 | — |
| 37 | LY6E | 1.867 | -1.483 | Discordant | 29 | 0.393 | 42.04 | R |
| 38 | DEFA4 | 1.046 | 3.454 | Concordant positive | 45 | 0.502 | 41.01 | P |
| 39 | MX1 | 1.640 | -1.848 | Discordant | 26 | 0.310 | 40.76 | — |
| 40 | HES4 | 1.251 | -1.845 | Discordant | 45 | 0.483 | 40.03 | — |
| 41 | ATP6V1C1 | 1.036 | 1.062 | Concordant positive | 43 | 0.466 | 39.96 | V |
| 42 | C1QB | 1.166 | 1.938 | Concordant positive | 30 | 0.353 | 39.51 | — |
| 43 | ACSL1 | 1.039 | 1.997 | Concordant positive | 33 | 0.321 | 36.61 | R |
| 44 | DHRS9 | 1.200 | 1.677 | Concordant positive | 40 | 0.360 | 36.24 | P;R |
| 45 | FOLR3 | 1.033 | 3.363 | Concordant positive | 34 | 0.290 | 34.83 | P |
| 46 | KCNJ15 | 1.047 | 1.092 | Concordant positive | 42 | 0.351 | 34.48 | — |
| 47 | TPST1 | 1.036 | 1.599 | Concordant positive | 48 | 0.459 | 32.83 | V |
| 48 | PPP1R3B | 1.023 | 1.104 | Concordant positive | 47 | 0.389 | 32.71 | P |
| 49 | SAP30 | 1.032 | 1.095 | Concordant positive | 49 | 0.453 | 30.99 | R |

Abbreviations and calculation: orthogonal score = mean(cross-cohort, scRNA specificity, ROC, protein); overall score = 0.5 × discovery composite + 50 × orthogonal score (0–100 scale). PPI metrics were reported only as supplementary network information and were not incorporated into the overall prioritization score. Therefore, SIGLEC1 and TRIM25 were not selected on the basis of PPI connectivity. Evidence codes: P, PPI rank ≤12; V, cross-cohort score ≥0.65; S, scRNA score ≥0.70; R, ROC score ≥0.80; Pr, protein score ≥0.70.
