# Supplementary material for: Single-cell profiling reveals context-dependent immune regulation shared by sepsis and systemic lupus erythematosus
Source: Front Immunol. 2026 Sep 3;17:1892194. doi: 10.3389/fimmu.2026.1892194 (PMC13581862; doi:10.3389/fimmu.2026.1892194)
Supplement: Supplementary file 3 [file Table2.docx]

**Supplementary Table 2. Comprehensive reconciliation of SIGLEC1 and TRIM25 expression patterns across discovery datasets, validation cohorts, meta-analysis reanalysis, single-cell RNA sequencing, and experimental models.**

| **Evidence tier** | **Gene** | **Dataset/source** | **Comparison** | **Sample type/model** | **Sample size** | **Direction** | **Effect size (95% CI)** | **P/FDR** | **Evidence note** |
| --- | --- | --- | --- | --- | --- | --- | --- | --- | --- |
| Discovery dataset | SIGLEC1 | CNP0002611 [S1] | Sepsis vs normal control | Not available | 23 vs 10 | Lower in sepsis | log2FC = -2.51 | FDR = 2.21E-03 | Discovery differential-expression result. |
| Discovery dataset | TRIM25 | CNP0002611 [S1] | Sepsis vs normal control | Not available | 23 vs 10 | Higher in sepsis | log2FC = 1.16 | FDR = 2.20E-07 | Discovery differential-expression result. |
| Discovery dataset | SIGLEC1 | GSE154851 [S2] | SLE vs healthy control | Whole blood | 38 vs 32 | Higher in SLE | log2FC = 1.61 | FDR = 1.31E-03 | Discovery differential-expression result. |
| Discovery dataset | TRIM25 | GSE154851 [S2] | SLE vs healthy control | Whole blood | 38 vs 32 | Higher in SLE | log2FC = 1.23 | FDR = 8.91E-04 | Discovery differential-expression result. |
| Sepsis validation | SIGLEC1 | GSE95233 [S3] | Adult sepsis at admission vs healthy control | Whole blood | 51 vs 22 | Lower in sepsis | Hedges' g = -0.77 (-1.28, -0.25) | P = 3.48E-03 | D01/admission samples only. |
| Sepsis validation | TRIM25 | GSE95233 [S3] | Adult sepsis at admission vs healthy control | Whole blood | 51 vs 22 | Lower in sepsis | Hedges' g = -1.22 (-1.76, -0.68) | P = 8.78E-06 | D01/admission samples only. |
| Sepsis validation | SIGLEC1 | GSE26378 [S3] | Pediatric septic shock vs normal control | Whole blood | 82 vs 21 | Higher in septic shock (NS) | Hedges' g = 0.29 (-0.19, 0.77) | P = 2.34E-01 | Harmonized single-cohort reanalysis. |
| Sepsis validation | TRIM25 | GSE26378 [S3] | Pediatric septic shock vs normal control | Whole blood | 82 vs 21 | Higher in septic shock | Hedges' g = 1.24 (0.73, 1.75) | P = 1.80E-06 | Harmonized single-cohort reanalysis. |
| Prognostic analysis | SIGLEC1 | GSE95233 [S3] | Non-survivor vs survivor at admission | Whole blood | 17 vs 34 | Lower in non-survivors (NS) | Hedges' g = -0.53 (-1.12, 0.06) | P = 8.03E-02 | D01/admission samples only. |
| Prognostic analysis | TRIM25 | GSE95233 [S3] | Non-survivor vs survivor at admission | Whole blood | 17 vs 34 | Higher in non-survivors (NS) | Hedges' g = 0.40 (-0.18, 0.99) | P = 1.77E-01 | D01/admission samples only. |
| SLE validation | SIGLEC1 | GSE72326 [S4] | SLE vs healthy control | Whole blood | 157 vs 20 | Higher in SLE | Hedges' g = 1.50 (1.01, 1.99) | P = 1.89E-09 | Independent whole-blood validation cohort. |
| SLE validation | TRIM25 | GSE72326 [S4] | SLE vs healthy control | Whole blood | 157 vs 20 | Higher in SLE | Hedges' g = 1.04 (0.56, 1.51) | P = 1.96E-05 | Independent whole-blood validation cohort. |
| Meta-analysis reanalysis | SIGLEC1 | GSE95233; GSE26378 [S5] | Across available sepsis clinical comparisons | Whole blood | Not pooled | Context-dependent | Not available (no pooled estimate) | Not available | Individual-cohort reanalysis only; one independent cohort was available per clinical comparison. |
| Meta-analysis reanalysis | TRIM25 | GSE95233; GSE26378 [S5] | Across available sepsis clinical comparisons | Whole blood | Not pooled | Context-dependent | Not available (no pooled estimate) | Not available | Individual-cohort reanalysis only; one independent cohort was available per clinical comparison. |
| scRNA-seq | SIGLEC1 | Dataset ID not recorded [S6-S7] | Cell-type localization analysis | Single-cell RNA sequencing | Not available | Localized to monocyte/macrophage populations | Descriptive single-cell localization | Not available | No quantitative logFC/FDR result file was found. |
| scRNA-seq | TRIM25 | Dataset ID not recorded [S6-S7] | Cell-type localization analysis | Single-cell RNA sequencing | Not available | Detected across multiple immune cell types | Descriptive single-cell localization | Not available | No quantitative logFC/FDR result file was found. |
| qPCR experimental model | SIGLEC1 | Experimental qPCR [S7-S8] | LPS-induced sepsis model vs Control | THP-1 macrophage model | 3 vs 3 | Higher in treated/model group | Fold change = 1.46 | Not available | LPS group; fold change derived from the three stored biological-replicate mean 2^-ΔΔCt values. No extractable P value was present. |
| qPCR experimental model | SIGLEC1 | Experimental qPCR [S7-S8] | OI-induced SLE model vs Control | THP-1 macrophage model | 3 vs 3 | Higher in treated/model group | Fold change = 1.95 | Not available | SLE group; fold change derived from the three stored biological-replicate mean 2^-ΔΔCt values. No extractable P value was present. |
| qPCR experimental model | TRIM25 | Experimental qPCR [S7-S8] | LPS-induced sepsis model vs Control | THP-1 macrophage model | 3 vs 3 | Higher in treated/model group | Fold change = 2.25 | Not available | LPS group; fold change derived from the three stored biological-replicate mean 2^-ΔΔCt values. No extractable P value was present. |
| qPCR experimental model | TRIM25 | Experimental qPCR [S7-S8] | OI-induced SLE model vs Control | THP-1 macrophage model | 3 vs 3 | Higher in treated/model group | Fold change = 2.13 | Not available | SLE group; fold change derived from the three stored biological-replicate mean 2^-ΔΔCt values. No extractable P value was present. |

**Notes.** Effect direction is defined as the first-named group minus the reference group. Discovery effects are log2 fold changes and are paired with Benjamini-Hochberg adjusted P values (FDR). Validation and prognostic effects are Hedges' g with 95% confidence intervals and two-sided P values. NS, not statistically significant (P >= 0.05).

The available sepsis evidence supports individual-cohort reanalysis, not a new pooled meta-analysis, because only one independent cohort was available for each clinical comparison. No quantitative scRNA-seq logFC/FDR file was found; those rows therefore report descriptive cell-type localization only. qPCR fold changes were computed from the three stored biological-replicate mean 2^-ΔΔCt values in the existing workbook, divided by the corresponding mean control value; no new hypothesis test was performed and P values were not available.
