# Supplementary material for: Single-cell profiling reveals context-dependent immune regulation shared by sepsis and systemic lupus erythematosus
Source: Front Immunol. 2026 Sep 3;17:1892194. doi: 10.3389/fimmu.2026.1892194 (PMC13581862; doi:10.3389/fimmu.2026.1892194)
Supplement: Supplementary file 4 [file Table3.docx]

**Supplementary Table S3. Disease-status coefficients before and after cell-composition adjustment**

| **Gene** | **Model** | **Beta** | **95% CI** | **P value** |
| --- | --- | --- | --- | --- |
| SIGLEC1 | Unadjusted | -2.442 | -3.792 to -1.092 | <0.001 |
| SIGLEC1 | Primary adjusted | -0.441 | -2.843 to 1.962 | 0.710 |
| SIGLEC1 | Full ssGSEA sensitivity | -0.347 | -2.767 to 2.074 | 0.771 |
| TRIM25 | Unadjusted | 1.163 | 0.869 to 1.457 | <0.001 |
| TRIM25 | Primary adjusted | 0.446 | 0.005 to 0.886 | 0.047 |
| TRIM25 | Full ssGSEA sensitivity | 0.399 | 0.001 to 0.798 | 0.050 |
