# Supplementary material for: Single-cell profiling reveals context-dependent immune regulation shared by sepsis and systemic lupus erythematosus
Source: Front Immunol. 2026 Sep 3;17:1892194. doi: 10.3389/fimmu.2026.1892194 (PMC13581862; doi:10.3389/fimmu.2026.1892194)
Supplement: Supplementary file 5 [file Table4.docx]

**Supplementary Table S4. Spearman correlations with monocyte and macrophage scores**

| **Gene** | **Cell score** | **rho** | **P value** | **N** |
| --- | --- | --- | --- | --- |
| SIGLEC1 | Monocyte score (clinical proxy) | -0.582 | <0.001 | 33 |
| SIGLEC1 | Macrophage score | -0.575 | <0.001 | 33 |
| TRIM25 | Monocyte score (clinical proxy) | 0.796 | <0.001 | 33 |
| TRIM25 | Macrophage score | 0.739 | <0.001 | 33 |
